# *Myelin basic protein* mRNA levels affect myelin sheath dimensions, architecture, plasticity, and density of resident glial cells

**DOI:** 10.1002/glia.24589

**Published:** 2024-07-18

**Authors:** Hooman Bagheri, Hana Friedman, Amanda Hadwen, Celia Jarweh, Ellis Cooper, Lawrence Oprea, Claire Guerrier, Anmar Khadra, Armand Collin, Julien Cohen‐Adad, Amanda Young, Gerardo Mendez Victoriano, Matthew Swire, Andrew Jarjour, Marie E. Bechler, Rachel S. Pryce, Pierre Chaurand, Lise Cougnaud, Dajana Vuckovic, Elliott Wilion, Owen Greene, Akiko Nishiyama, Anouk Benmamar‐Badel, Trevor Owens, Vladimir Grouza, Marius Tuznik, Hanwen Liu, David A. Rudko, Jinyi Zhang, Katherine A. Siminovitch, Alan C. Peterson

**Affiliations:** ^1^ Department of Human Genetics McGill University Montreal Quebec Canada; ^2^ Department of Physiology McGill University Montreal Quebec Canada; ^3^ Department of Pharmacology & Therapeutics McGill University Montreal Quebec Canada; ^4^ Integrated Program in Neuroscience McGill University Montréal Quebec Canada; ^5^ Université Côte d'azur, LJAD, CNRS UMR7351 Nice France; ^6^ Institute of Biomedical Engineering, Ecole Polytechnique de Montreal Montreal Quebec Canada; ^7^ Department of Cell and Developmental Biology State University of New York Upstate Medical University Syracuse New York USA; ^8^ Department of Neuroscience and Physiology State University of New York Upstate Medical University Syracuse New York USA; ^9^ Department of Chemistry Université de Montréal Montreal Quebec Canada; ^10^ Department of Chemistry and Biochemistry Concordia University Montreal Quebec Canada; ^11^ Department of Physiology and Neurobiology University of Connecticut Storrs Connecticut USA; ^12^ Institute for Systems Genomics, University of Connecticut Storrs Connecticut USA; ^13^ The Connecticut Institute for Brain and Cognitive Sciences, University of Connecticut Storrs Connecticut USA; ^14^ Department of Neurobiology Research Institute for Molecular Medicine, University of Southern Denmark Odense Denmark; ^15^ McConnell Brain Imaging Centre, Montreal Neurological Institute and Hospital Montreal Quebec Canada; ^16^ Department of Neurology and Neurosurgery McGill University Montreal Quebec Canada; ^17^ Department of Biomedical Engineering McGill University Montreal Quebec Canada; ^18^ Department of Medicine University of Toronto Toronto Ontario Canada; ^19^ Department of Immunology University of Toronto Toronto Ontario Canada; ^20^ Mount Sinai Hospital, Lunenfeld‐Tanenbaum and Toronto General Hospital Research Institutes Toronto Ontario Canada; ^21^ Gerald Bronfman Department of Oncology McGill University Quebec Canada

**Keywords:** astrocyte, hypermyelination, hypomyelination, *Mbp* transcription, microglia, myelin basic protein, myelin elaboration and plasticity, myelin sheath thickness and length, oligodendrocyte and oligodendrocyte progenitor cell

## Abstract

Myelin Basic Protein (MBP) is essential for both elaboration and maintenance of CNS myelin, and its reduced accumulation results in hypomyelination. How different *Mbp* mRNA levels affect myelin dimensions across the lifespan and how resident glial cells may respond to such changes are unknown. Here, to investigate these questions, we used enhancer‐edited mouse lines that accumulate *Mbp* mRNA levels ranging from 8% to 160% of wild type. In young mice, reduced *Mbp* mRNA levels resulted in corresponding decreases in *Mbp* protein accumulation and myelin sheath thickness, confirming the previously demonstrated rate‐limiting role of *Mbp* transcription in the control of initial myelin synthesis. However, despite maintaining lower line specific *Mbp* mRNA levels into old age, both MBP protein levels and myelin thickness improved or fully normalized at rates defined by the relative *Mbp* mRNA level. Sheath length, in contrast, was affected only when mRNA levels were very low, demonstrating that sheath thickness and length are not equally coupled to *Mbp* mRNA level. Striking abnormalities in sheath structure also emerged with reduced mRNA levels. Unexpectedly, an increase in the density of all glial cell types arose in response to reduced *Mbp* mRNA levels. This investigation extends understanding of the role MBP plays in myelin sheath elaboration, architecture, and plasticity across the mouse lifespan and illuminates a novel axis of glial cell crosstalk.

## Main Points

- *Mbp* mRNA level limits myelin elaboration but that relationship changes with age.
- *Mbp* mRNA reduction affects sheath thickness, length, structure, and adult plasticity.
- *Mbp* reduction precipitates a marked increase in the density of all glial lineages.

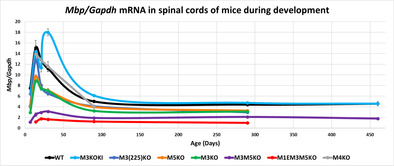

## 1 INTRODUCTION

The acquisition of myelin during vertebrate evolution conferred fundamental enhancements to nervous system function (Castelfranco & Hartline, 2016; Zalc et al., 2008). While supporting accelerated and energy efficient conduction of action potentials, myelin sheaths also provide trophic and metabolic support to axons (Cohen et al., 2020; Simons & Nave, 2015). Their radial and longitudinal dimensions correlate with the caliber of the axons they surround (Almeida et al., 2011; Hess & Young, 1949; Waxman & Sims, 1984), but also demonstrate plasticity in response to circuit activity that may serve to synchronize synaptic inputs (Pajevic et al., 2023). Demonstrating the critical role myelin plays in normal nervous system function, inherited, or acquired myelin disruption underlies multiple debilitating diseases (Depp et al., 2023; Stadelmann et al., 2019). Further, when the generation of new myelin is disrupted experimentally in mice, motor task learning, memory, and behavior are perturbed (Bacmeister et al., 2020; Bercury & Macklin, 2015; Bergles & Richardson, 2015; Bonetto et al., 2021; Pan et al., 2020; Steadman et al., 2020).

Mutations in genes encoding myelin components have illuminated the specific roles played by major myelin proteins (Miyata, 2019). Notable among these are spontaneous and experimentally induced mutations in the *Golli/Mbp* locus that affect *Myelin basic protein* (*Mbp*) expression (Bagheri et al., 2020; Popko et al., 1987; Readhead et al., 1987; Roach et al., 1985; Roch et al., 1987; Shine et al., 1992). Shiverer mice are null for *Mbp*, and while their oligodendrocytes are capable of ensheathing axons with a few layers of plasma membrane, these typically fail to compact into dense myelin sheaths (Inoue et al., 1981; Roach et al., 1985; Rosenbluth, 1980). In addition, transgenic preparations revealed a close correlation between the volume of elaborated myelin and levels of *Mbp* mRNA accumulation, thus implicating a rate‐limiting role for *Mbp* imposed at the transcriptional level (Shine et al., 1992). Most recently, experimentally induced deletion of *Mbp* in mature mice demonstrated that maintenance of pre‐formed sheaths also requires continuous MBP synthesis (Meschkat et al., 2022). Thus, MBP plays an essential role in both myelin elaboration and maintenance. Additionally, GOLLI, the other product of the *Golli/Mbp* locus, accumulates in multiple lineages, both within and outside the CNS, where it plays a role in Ca^2+^ signaling (Campagnoni et al., 1993; Cheli et al., 2016; Feng, 2007; Landry et al., 1996; Paez et al., 2009; Paez et al., 2012; Paez & Lyons, 2020). Notably, in mice that lack or overexpress GOLLI, myelination in the visual system is perturbed (Jacobs et al., 2005; Jacobs et al., 2009).

Here we investigated the consequences of altered *Mbp* mRNA expression using mouse models in which the enhancers that control the transcription of the *Golli/Mbp* locus have been edited or deleted. We showed previously that such edits perturb both *Golli* and *Mbp* mRNA accumulation resulting in different line‐specific levels in the CNS that are maintained up to P90 (Bagheri et al., 2020; Dib et al., 2011). Here, we extend such characterization into old age and include a new line in which M3 was exchanged with a multimerized M3 sub‐sequence leading to higher‐than‐normal *Golli/Mbp* mRNA accumulation. Combined, these edits result in a 6‐member panel in which *Mbp* mRNA levels range in a graded fashion between 8% and 160% of wild type (WT), providing novel opportunities to investigate the role of MBP in myelin elaboration and maintenance. We provide insight into the relative role of transcriptional and post‐transcriptional control of MBP protein production, the relationship between *Mbp* mRNA levels and sheath thickness and length, and the consequences of reduced *Mbp* mRNA levels on sheath architecture. Finally, we observed an unanticipated increase in the density of all glial cell types in response to graded hypomyelination exposing a novel axis of glial crosstalk.

## 2 METHODS

### 2.1 Animals

All experiments were carried out in accordance with the guidelines of the Canadian Council on Animal Care. Protocol number 215–7668 approved by the McGill University DOW Facilities Animal Care Committee. Animals were on a 12 h light/dark cycle and a 2920X Teklad diet from Envigo.

Mouse lines were generated as described in Bagheri et al., 2020. The M3KOKI allele is deleted of M3 (chr18:82,567,315‐82,568,165 GRCm39/mm39) and is replaced by an insertion of three full and one partial copy of a ~ 105 bp sequence that originates from the core of M3. All analysis was performed on mice derived from litters of 6–8 pups. Pups were weaned between P24–P28 and caged in groups of 4 to 5 of the same sex.

### 2.2 qRT‐PCR and mRNA analysis

Samples from homozygous mice (typically *n* = 6) of both genders were obtained at the ages indicated (Tables S1 and S2). Mice were anesthetized with a lethal dose of Avertin, and samples of sciatic nerve, cervical spinal cord, optic nerve, and thymus were collected into RNAlater solution (Ambion) according to manufacturer's instructions and stored at −20°C. RNA extraction was achieved using Trizol (Life Technologies) and a Qiagen RNeasy MinElute Cleanup kit according to manufacturer's instructions. qRT‐PCR was as described in Bagheri et al., 2020. Statistical analysis was performed using GraphPad Prism V9.5.1, one‐way ANOVA and Dunnett's multiple comparisons or Brown‐Forsythe and Welch ANOVA and Dunnett's T3 multiple comparisons tests.

### 2.3 Western blot

Homozygous mice (*n* = 3) of mixed genders at P14 and P90 were anesthetized with a lethal dosage of Avertin and samples of cervical spinal cord were immediately frozen on dry ice. Extracts were obtained using RIPA lysis buffer and WB was performed using anti‐GAPDH (Cell Signaling #2118), anti‐2',3'‐Cyclic‐nucleotide 3'‐phosphodiesterase (CNPase) (Cell Signaling #5664), and anti‐MBP (Sigma‐Aldrich MAB386). One sample from each genotype and age was run on each gel resulting in three sets of comparisons per age. MBP and CNPase values were first normalized to GAPDH and then to wild type (WT) (100%) of the same set.

### 2.4 Imaging mass spectrometry peptide and cholesterol analysis

Spinal cord samples from homozygous mice (*n* = 5) of both genders at P90 were collected as for mRNA analysis. Five tissue microarrays (TMAs) each including one sample from each of the eight genotypes were built by encasing spinal cords in 1.5% carboxymethylcellulose. Cryostat cross sections (Thermo Fisher Scientific Microm HM550) were cut at 12 μm thickness and thaw‐mounted on indium‐tin oxide (ITO)‐coated glass slides (Delta Technologies, Loveland, CO) and desiccated for 30 min before further sample preparation. All TMAs were analyzed in triplicate.

For peptide analysis, sections were washed 30s 70% ethanol, 30s 100% ethanol, 2 min Carnoy's solution (60% ethanol, 30% chloroform and 10% glacial acetic acid), 30s 100% ethanol, 30s HPLC‐grade water, 30s 100% ethanol prior to trypsin digestion (Gessel et al., 2015). Twenty microgram of pierce trypsin protease was dissolved in 250 μL of 100 mM ammonium bicarbonate and deposited using an M3 TM‐sprayer (HTX Technologies, Chapel Hill, NC, USA) using the following parameters: 750 mm/min velocity, track spacing 2 mm, 30°C, 10 psi, pattern VV, 40 mm nozzle height, flow rate of 7.5 μL/min, and 7 passes. The samples were then incubated in a hydration chamber overnight at 37°C and desiccated prior to matrix application. α‐Cyano‐4‐hydroxyciannamic acid matrix in 50:50 acetonitrile: water, 0.1% trifluoroacetic acid was deposited on the samples using the M3 TM‐sprayer at 1200 mm/min velocity, track spacing 3 mm, 65°C, 10 psi, pattern VV, 40 mm nozzle height. Approximate matrix thickness was 0.001667 mg/mm^2^.

Imaging mass spectrometry (IMS) was performed on a MALDI TOF/TOF ultrafleXtreme mass spectrometer equipped with SmartBeam II Nd:YAG/355 nm laser operating at 2000 Hz (Bruker Daltonics, Billerica, MA). IMS data was acquired in positive ion reflectron mode under optimized delayed extraction conditions using 300 shots per pixel with a spatial resolution of 50 μm in the 700–5000 Da mass range. To estimate protein abundance, a reporter tryptic peptide from the *Mbp* sequence was chosen at m/z 2141.4 [M + H]^+^. The identity of this peptide was confirmed by sequencing using MS/MS performed in positive polarity on the MALDI TOF/TOF ultrafleXtreme mass spectrometer (data not shown).

For cholesterol analysis, spinal cord cross sections were analyzed by silver‐assisted laser desorption ionization IMS as described in Dufresne et al. (Dufresne et al., 2013). Silver deposition was performed using a Cressington 308R sputter coater (Ted Pella Inc., Redding, CA, USA). These parameters (argon partial pressure of 0.02 mbar and current of 80 mA for 30s) give a silver layer of approximately 28 nm thickness. IMS data was acquired in positive ion reflectron mode under optimized delayed extraction conditions using 500 shots per pixel with a spatial resolution of 50 μm in the 250–1100 Da mass range. For cholesterol abundance, the peak at m/z 493.2 [M + ^107^Ag]^+^ was considered.

IMS data was visualized in FlexImaging 4.1 software (Bruker Daltonics) and exported into imzML format prior to importation into R (v 4.2.3). Using the Cardinal Package (v 3.2.1), the baseline was reduced, followed by normalization by total ion current (TIC). The average intensity of each pixel for masses of interest were then extracted and combined to generate averages for each region of interest. The most and least intense pixels (top and bottom 10% of the data for each region of interest) were excluded from calculations to reduce variability due to outliers and changes in histology throughout the white matter. Using GraphPad Prism V9.5.1, one‐way ANOVA and Dunnett's multiple comparisons test were performed, and plots generated.

### 2.5 Liquid chromatography‐mass spectrometry cholesterol analysis

Individual spinal cords from homozygous mice (typically *n* = 5) of both genders at P90 were thawed on ice and weighed. Extraction solvent (methanol/isopropanol/water, 3/5/2, v/v/v, LC–MS grade) was added keeping a constant tissue to solvent ratio (0.1 g/ 1 mL). Samples were homogenized using a Precellys (Bertin Technologies, 6500 rpm, 4 cycles of 15 s with 30 s of break) at 15–16°C using liquid nitrogen cooling, vortexed for 5 min, incubated on ice for 15 min and centrifuged at 25000 × g for 10 min at 4°C. The resulting supernatant was diluted 1:40 with the extraction solvent prior to analysis. The LC–MS analysis was performed using an untargeted lipidomics method on an Agilent 1100 LC system coupled with an LTQ‐Velos Orbitrap (Thermo Fisher Scientific) (Bowden et al., 2017). Chromatographic separation utilized a Waters CSH C18 column (130 Å, 2.5 μm, 2.1 × 75 mm) with a 0.25 mL/min flow rate and column temperature of 55°C. The mobile phase was composed of A: water/methanol (60/40, v/v) with 10 mM of ammonium acetate and B: isopropanol/methanol (90/10) with 10 mM of ammonium acetate. The gradient program was as follows: 0–2 min 20% B; 2–4 min 20%–30% B; 4–25 min 30%–80% B; 25–35 min 80%–85% B, 35–38 min 85%–95% B, 38–41 min 95% B, 41–41.1 min 95%–20% B; 41.1–50 min 20% B. The injection volume was 10 μL. Mass spectrometry acquisition was performed in positive electrospray mode, with full MS1 scan mass range of 345–1400 m/z at 60,000 resolving power with MS/MS analysis using top eight collision induced dissociation data‐dependent acquisition in ion trap at nominal resolution. The identification of cholesterol was confirmed using authentic 5 μg/mL standard (Sigma Aldrich), and relative quantification was performed using m/z 369.3531 with 5 ppm extraction window using XcaliburTM 2.2 software (Thermo Fisher Scientific). GraphPad Prism V9.5.1, one‐way ANOVA and Dunnett's multiple comparisons tests were performed, and plots generated.

### 2.6 Spinal cord area measurements

Homozygous mice (*n* = 3–4) at P30 were anesthetized with a lethal dose of Avertin and perfused transcardially with 10 mL of 1X phosphate buffer saline (PBS) followed by 10 mL of 4% PFA (FDNeuroTechnologies) at 1 mL/min. Cervical spinal cords were post‐fixed in 4% PFA for 2 h and flash frozen in liquid nitrogen cooled isopentane. Twelve micrometer of thick sections were obtained at the C5‐C6 level and imaged with a Zeiss Axio Observer Microscope at 20X using phase contrast. Ventrolateral and dorsal column white and gray matter were manually outlined for area calculation using Zen Software. Nine images were obtained for each genotype (3 sections from 3 spinal cords). Multiple unpaired *t*‐test was performed, and plots were generated using GraphPad Prism V9.5.1.

### 2.7 MRI

Homozygous mice (*n* = 3) were lethally anesthetized with Avertin and perfused transcardially with 10 mL of HBSS followed by 10 mL of 2.5% glutaraldehyde, 4% PFA at 1 mL/min as described in Weil et al. (Weil et al., 2019). Brains and spinal cords were post‐fixed overnight, and then washed in 1X PBS daily for 7 days. Imaging was performed using the Bruker (Billerica, MA, USA) Pharmascan 7 Tesla, Pre‐Clinical MRI system. Multi‐echo gradient echo, *T*_2_*‐weighted image volumes (2 millisecond first echo time and 2 millisecond echo spacing; 24 echoes; matrix size of 165 × 201 × 165; 100 um^3^ isotropic resolution) were acquired using a 3D multi‐echo gradient‐recalled echo (mGRE) sequence with a bipolar readout. For each imaging session, five spinal cords were randomly selected and distributed within a custom‐designed receptacle. All spinal cords were imaged within three imaging sessions. Following reconstruction, the image volumes were corrected for bipolar gradient phase offset (Lu et al., 2008) and Gibb's ringing (Kellner et al., 2016). A binary mask for the volume corresponding to C2–C7 of each spinal cord was produced using Otsu's median thresholding method in addition to manual adjustment. The 24‐echo mGRE image volume was decomposed into components corresponding to myelin water, intra/extracellular (IE) water, and free water pools using a self‐labeled encoder decoder network (Liu et al., 2022). The myelin water fraction (MWF) was calculated as the ratio of the myelin water pool to the sum of the myelin, IE, and free water pools as previously described (Liu et al., 2022). The MWF was analyzed within the whole spinal cord corresponding to C2–C7. Using GraphPad Prism V9.5.1, one‐way ANOVA and Dunnett's multiple comparisons test were performed to evaluate differences in MWF between each genotype. Scattered plots were generated representing % MWF for each genotype.

### 2.8 Electron microscopy and g‐ratio analysis

Mice homozygous for each allele were lethally anesthetized with Avertin, perfused transcardially and spinal cord and brain processed as described in Weil et al. (Weil et al., 2019). For qualitative assessment, image from cervical level spinal cord cross sections of gracile and cuneate (GC), ventromedial (VM), and corticospinal tracts (CST) were obtained. Images also were obtained from the body of the corpus callosum (CC) at midline from 1 mm thick longitudinal brain sections from which samples containing the callosal body were recovered and orientated to yield cross sections.

Quantitative analysis of myelin sheath thickness was performed on images obtained as non‐overlapping tiles at 2900x–9300x with fibers segmented using Axondeepseg (Zaimi et al., 2018). Only the subset of fibers with uniformly compact sheaths closely associated with greater than 90% of the axon circumference were included in the g‐ratios analysis. Two mice from each of the M3KO and M3KOKI lines were analyzed to estimate potential inter‐animal variability and, as such variation was negligible, g‐ratio analysis was performed on three different tracts from single mice of seven different genotypes at three different ages. Axon and axon plus myelin diameters were calculated from perimeter measurements. Graphs were generated using Microsoft Excel.

To determine if axon diameters were affected by their myelination status, axons in images of the GC tracts obtained from P30 and P450 mice were selected without regard to myelin morphology, an approach resulting in a population of fibers larger than that selected for g‐ratio calculations. All complete axon profiles in the available sets of 20 to 30 non‐overlapping 4800x images from each genotype were included in the analysis.

For g‐ratio comparisons, analysis of covariance (ANCOVA) models was performed using R, version 4.3.0, to investigate the possible difference in mean g‐ratios between genotypes, after adjusting for axon diameter. Axon diameter range was divided into four bins and ANCOVA was performed to compare each bin with WT. The assumption of homogeneity of linearity for the ANCOVA model was checked by introducing and testing an interaction effect term between genotype and axon diameter. The assumptions of normality and equal variances of errors, as well as the presence of possible outliers, were explored with graphical analysis of residuals. Differences in mean g‐ratio were reported with bootstrap 99% confidence intervals. Because the experiment was performed using one mouse per genotype, the assumption of independence of observations is violated and the standard errors of the estimates may be biased down and, *p*‐values and CIs may therefore be unreliable.

### 2.9 Sheath lengths in spinal cord white matter

Homozygous mice (*n* = 4–5) at P30‐31 were anesthetized with a lethal dose of Avertin and perfused transcardially with 10 mL of 1X PBS followed by 10 mL of 4% PFA (FDNeuroTechnologies) at 1 mL/min. Spinal cord and brain samples were collected and post‐fixed in 4% PFA (FDNeuroTechnologies) for 30 min, washed with PBS and stored in PBS at 4°C before the next step.

Ventral and lateral white matter from cervical and thoracic cords were cut into strips and teased apart using acupuncture needles (32 gauge, 0.25 mm diameter, 25 mm length) onto SuperFrost Plus slides (VWR) as described (Jarjour & Sherman, 2019). Slides were washed with PBS, pH 7.4, then incubated in blocking solution (3% normal donkey serum, 2% bovine serum albumin, 0.1% TritonX‐100 in PBS). Nerve fibers were incubated with primary antibodies diluted in blocking solution overnight at room temperature. Primary antibodies were: NFH 1:10,000 (Biolegend #822601, RRID: AB_2564859), MAG 1:100 (Santa Cruz Biotechnology sc‐9544, RRID: AB_670102), Caspr 1:500 (Abcam #ab34151, RRID: AB_869934). Slides were washed with blocking solution then incubated 1 h at room temperature in secondary antibodies donkey anti‐rabbit AlexaFluor 488, anti‐goat AlexaFluor 633 (Invitrogen #A21206, #A21082, RRID: AB_2535792, AB_2535739) at 1:1000, and donkey anti‐chicken DyLight 405 (Jackson ImmunoResearch 703–475–155, RRID:AB_2340373) at 1:500 diluted in blocking solution. Fibers were washed with blocking solution, then PBS, and mounted with Fluoromount G (Southern Biotech) and a glass coverslip.

Tiled images were obtained using a Leica SP8 confocal microscope 20x/NA 0.7 objective. Composite z‐stack max projections of blinded images were used for analysis in FIJI ImageJ (Schindelin et al., 2012). Complete myelin sheaths were defined as a MAG overlapping NFH with Caspr labeled ends. Sheath lengths were measured from Caspr band to Caspr band. Axon diameters were measured adjacent to the paranode. In regions after the paranode where bulging of myelin sheath was seen adjacent to the paranode, diameter measurements were taken below the uneven section. More than 40 sheaths were measured per mouse of each genotype: M3KO (*n* = 4), M5KO (*n* = 5), M3KO,389bpKI (*n* = 5), and WT mice (*n* = 5). For each genotype, the total number of fibers measured was > 49 (small diameter), > 200 (medium diameter), and > 70 (large diameter). Sample size was pre‐determined to give power > 0.8, sensitive to up to 20% changes in myelin sheath length.

Using GraphPad Prism V9.5.1, one‐way ANOVA with Tukey's post hoc analysis was used to determine significance. For representative confocal images in figures, color balance was adjusted to see all channels in the merge.

### 2.10 Sheath lengths and number in cortex

Analysis of motor cortex myelin sheaths was performed on 4–5 homozygous mice of each genotype at P30–31 or P85, modified from Swire et al (Swire et al., 2019). After transcardial perfusion with 4% PFA, brains were collected and postfixed in 4% PFA. Coronal sections of 200 μm thickness were made using a vibrating blade microtome (IMEB, Vibratome 1000 PLUS), collected from bregma +1.18 mm to bregma +1.10 mm anterior–posterior. Brain sections were heated 20 min in 11.4 mM trisodium citrate (RPI chemicals) pH 6 with 0.05% Tween20 for antigen retrieval. Sections were blocked with 10% goat serum (Sigma‐Aldrich) 0.5% TritonX‐100 in PBS for 2 h. Immunolabeling was made with 1:2000 mouse anti‐CNPase (Atlas AMAb91068), 1:250 rabbit anti‐Caspr (Abcam ab34151), and 1:250 rat anti‐MBP (BioRad AbD Serotec MCA409S) diluted in blocking buffer for 24 h at 4°C. After wash steps, slices were incubated 24 h at 4°C in goat Alexa Fluor‐conjugated secondary antibodies (ThermoFisher Scientific) diluted 1:1000 in blocking buffer: anti‐rat 555, anti‐mouse 488, and anti‐rabbit 633. PBS washes were followed by a 2 h incubation with Hoechst (Cayman Chemical). Slices were mounted onto a glass slide with Fluoromount‐G and cover glass.

Quantification was blinded and performed in the same layer II‐III cortical region for both brain hemispheres. For each mouse, 6–7 fields with 0.4 μm step z‐series images were acquired with a Leica SP8 microscope and a 40x/NA1.1 objective. The SNT plug‐in of ImageJ (version 1.53f51, Wayne Rasband, NIH, USA) software (Longair et al., 2011) was used to facilitate myelin sheath length measurements in 3D. CNPase staining was used trace processes from a cell body connected to myelin sheaths (Caspr‐flanked MBP^+^ stretch). Single cells that had all processes and myelin sheaths fully captured in the 200 μm thick section were used for analysis of sheath number per individual oligodendrocyte. Seven individual cells per mouse were analyzed. Myelin sheath length defined by CNPase staining between flanking Caspr bands was measured for myelin sheaths completely contained within the vibratome section. A minimum of 204 myelin sheath lengths were measured per animal. Statistical power and sample size were calculated with the G*Power (version 3.1.9.7, Heinrich Heine University Düsseldorf) software. Power of 0.8 and alpha 0.05, with an effect size of 5 μm was used. Cortical sheath lengths were log normal, therefore comparisons between groups were performed on the mean log sheath lengths with one‐way ANOVA and Tukey post‐hoc testing.

### 2.11 Cell density analysis

Homozygous mice were lethally anesthetized with Avertin and perfused with 4% paraformaldehyde containing 0.1 M lysine and 0.01 M sodium metaperiodate in 0.1 M sodium phosphate buffer, pH 7.2 at 1 mL/min. Spinal cords were recovered and postfixed for 1 h at 4°C. After four rinses in sodium phosphate buffer, the tissues were cryoprotected in 30% sucrose in phosphate buffer, embedded and frozen in O.C.T. Compound (Sakura Finetek, Tissue‐Tek 4583), and stored at −80°C. Transverse sections of the spinal cord were cut at 20 μm thickness on a Cryostat (Leica CM3050‐S) and stored at −20°C.

For immunofluorescence labeling, sections were rinsed in PBS and blocked and permeabilized with 5% bovine serum albumin (BSA) and 0.3% Triton‐X‐100 in PBS for 1 h at room temperature, incubated at 4°C overnight in primary antibody solution containing goat anti‐mouse PDGFRa (1:1000, R&D Systems, #AF1062, RRID: AB_2236897), rabbit anti‐GST‐p (1:500, MBL International, #312, RRID:AB_591792), and mouse monoclonal anti‐Quaking 7 antibody clone CC1 (1:200, Millipore, #OP80, RRID:AB_2057371) diluted in the blocking buffer. After a 3x PBS rinse, they were incubated in a secondary antibody solution containing Alexa 488‐conjugated donkey anti‐rabbit (1:1000, ThermoFisher, #A21206, RRID:AB_2535792), Cy3‐conjugated bovine anti‐goat (1:500, Jackson ImmunoResearch, #805–165‐180, RRID:AB_2340880), and Alexa 647‐conjugated donkey anti‐mouse antibodies (1:200, ThermoFisher, #A31571, RRID:AB_162542) was added for 1 h at room temperature in the dark. Following a 3x PBS rinse, sections were mounted using 25–50uL of ProLong™ Gold Antifade Mountant with DAPI (ThermoFisher, #P36931). Slides were cured for at least 24 h and stored at −20°C. Confocal images were acquired using a Leica SP8 Spectral Confocal Microscope and Leica LAS X software. Quantification of labeled cells in the dorsal column of the cervical spinal cord were performed using the ImageJ Cell Counter Plugin. Using the “Freehand selections” tool, the border of the dorsal column of the cervical spinal was outlined, and the area was acquired using the ImageJ “Measure” feature. The readout in square microns was converted to square millimeters before continuing with analysis. Immunoreactive cells within the defined area were marked and counted using the ImageJ Cell Counter Plugin. A minimum of three animals for each genotype and 400 cells for each dorsal column was included in the analysis. Using GraphPad Prism V9.5.1, one‐way ANOVA and Dunnett's multiple comparisons test were performed, and plots were generated.

## 3 RESULTS

### 3.1 Mutated enhancers affect *Golli* and *Mbp* mRNA accumulation throughout the mouse lifespan

**FIGURE 1:**
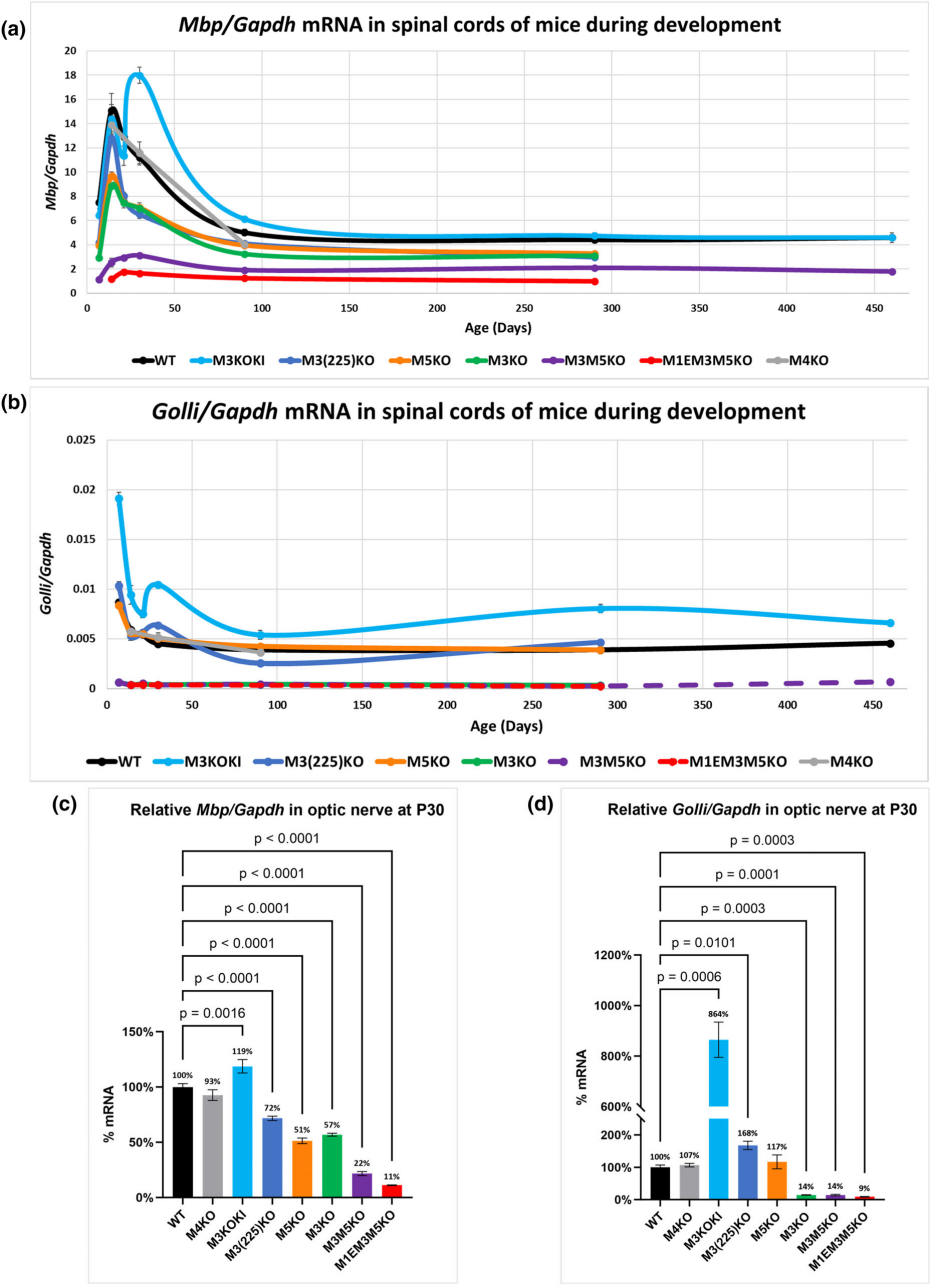
*Myelin basic protein* (*Mbp*) and *Golli* mRNA accumulation in enhancer‐edited mice. (a) Relative *Mbp/Gapdh* in spinal cord (*n* = 4–11). (b) Relative *Golli/Gapdh* in spinal cord (*n* = 4–11). Differences in line‐specific *Golli/Mbp* mRNA levels are largely maintained throughout life. (c) Relative *Mbp/Gapdh* in optic nerve at P30 (*n* = 5–6). (d) Relative *Golli/Gapdh* in optic nerve at P30 (*n* = 5–6). Relative *Golli/Mbp* mRNA levels are similar in both optic nerve and spinal cord. *p*‐values are calculated using one‐way ANOVA in (a–c) and Brown‐Forsythe and Welch ANOVA in (d) followed by Dunnett's T3 multiple comparisons tests. All data are presented as mean, and error bars indicate SEM.

As shown previously, mice bearing different enhancer mutations accumulated line‐specific levels of *Mbp* mRNA in spinal cord that typically followed the normal developmental expression program through P90 (Bagheri et al., 2020). An exception was the newly derived M3KOKI allele that drove expression to levels equal to or higher than WT (160% at P30 and 120% at P90) (Figure 1a and Supplementary Table **S1**). In this experimentally derived allele, M3 is replaced by a multimerized M3 sub‐sequence (Supplementary Figure **S1**) that supports two *Mbp* mRNA accumulation peaks, one typical of WT prior to weaning and a second shortly after (Figure 1a), likely exposing a change in the transcription factor repertoire expressed in oligodendrocytes during primary myelin elaboration and postweaning myelin maintenance. mRNA analysis was extended to P290 in all lines and up to P460 for WT, M3M5KO and M3KOKI mice (Figure 1a and Supplementary Table S1). The inter‐line accumulation differences observed for P90 spinal cord largely were maintained through to the oldest age examined. Further, relative *Mbp* mRNA accumulation in both spinal cord and optic nerve at P30 was similar (Figure 1c).

The M3 enhancer accounts for ~40% of the *Mbp* mRNA accumulation in oligodendrocytes. Exceptionally, it also accounts for ~90% of *Golli* mRNA accumulation in multiple lineages both within and beyond the nervous system (Bagheri et al., 2020; Dib et al., 2011). In contrast, mice bearing the substituted M3KOKI allele accumulated higher *Golli* levels in spinal cord (140%–230%), normal levels in thymus (107% of WT, data not shown) and exceedingly high levels in P30 optic nerve (864% of WT) (Figure 1b,d and Table S2). We investigated the possible effects of *Golli* disruption on the application of these models. Notably, mice entirely null for *Golli* show delayed myelination and hypomyelination that improves with age (Jacobs et al., 2005). Importantly, myelin disruption in *Golli* null mice was restricted entirely to the optic system suggesting that our analysis of spinal cord, a domain where the complete absence of *Golli* has no obvious effect, should not be affected by any reduction in *Golli* mRNA accumulation. Of most relevance, mice in the M3KO and M5KO lines accumulate the same reduced level of *Mbp* mRNA but, *Golli* mRNA is decreased only in M3KO mice. As both M3KO and M5KO mice demonstrated the same myelin phenotypes, reduced *Golli* mRNA accumulation consequent upon the M3KO deletion has no demonstrable effect on the spinal cord myelin examined in the present study.

### 3.2 The relationship between the accumulated levels of *Mbp* mRNA and MBP protein changes with age

Early pioneering investigations showed that mice bearing combinations of different alleles that included an *Mbp* encoding transgene, the *Mbp* null shiverer and myelin deficiency (*mld*) alleles accumulated *Mbp* mRNA in the CNS over a wide range that correlated closely with MBP protein levels in the brain (Popko et al., 1987; Shine et al., 1992). To determine if a similar correlation exists in the spinal cord, MBP protein accumulation in the different lines of enhancer‐edited mice was measured by Western Blot (WB) and IMS and compared to *Mbp* mRNA levels.

**FIGURE 2:**
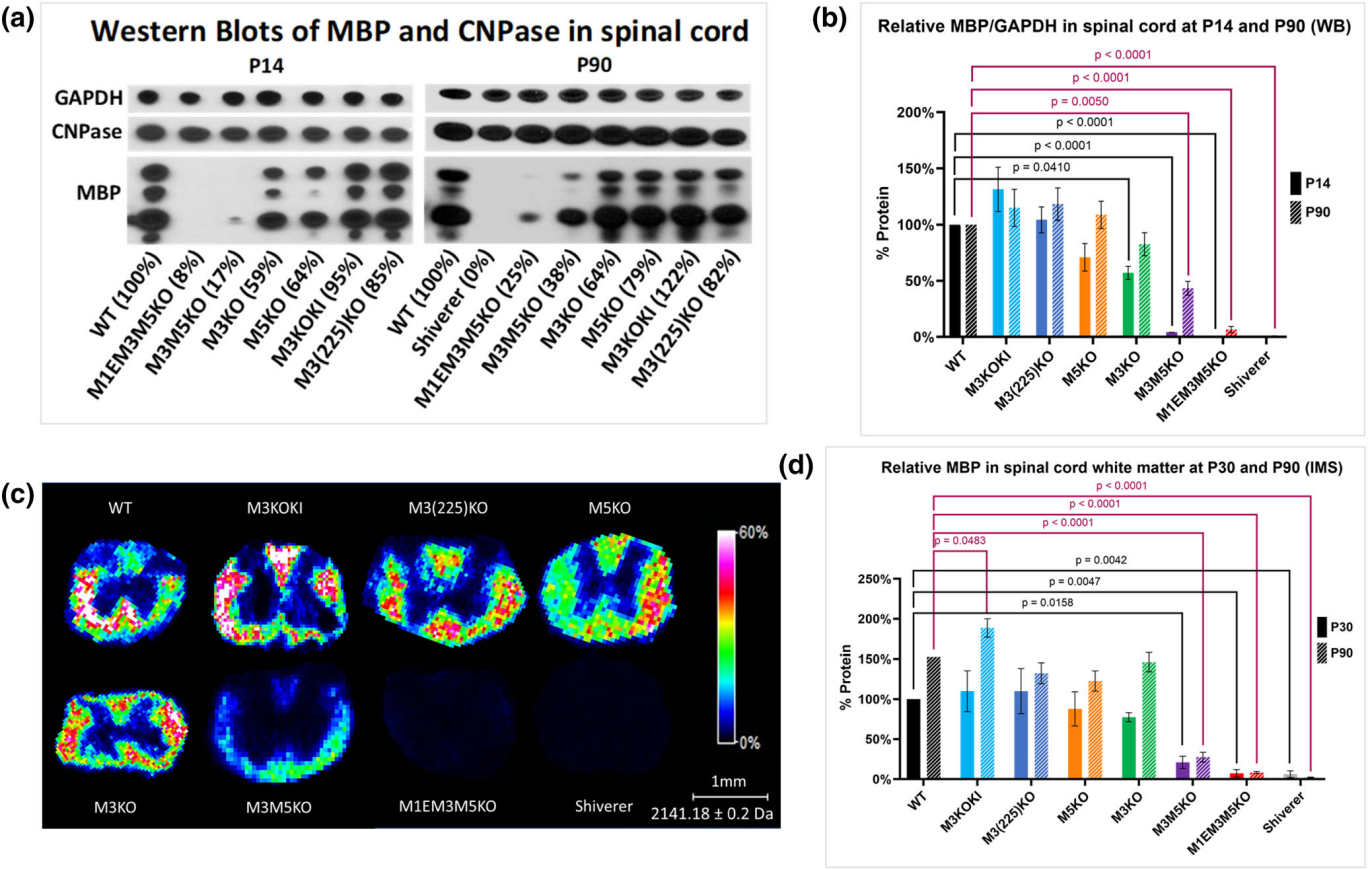
Relative Myelin basic protein (MBP) levels in spinal cord of enhancer‐edited mice as determined by Western blot (WB) and imaging mass spectrometry (IMS). (a), Western blots of MBP and CNPase in spinal cord at P14 and P90. Relative mRNA values at P14 and P90 are indicated as a percentage of WT. (b), MBP/GAPDH in spinal cord at P14 and P90 measured by WB with all values normalized to WT of the same age (*n* = 3 for each genotype and age). (c), MBP distribution within the cervical spinal cord measured by IMS at P90. (d), Relative MBP in spinal cord WM at P30 (*n* = 3) and P90 (*n* = 5) measured by IMS with all values normalized to WT at P30. MBP protein accumulation increases with age. *p*‐values are calculated using one‐way ANOVA and Dunnett's multiple comparisons test. All data are presented as mean, and error bars indicate SEM.

For WB analysis, samples from the cervical spinal cord were obtained at P14, near the normal pre‐weaning peak of *Mbp* mRNA accumulation, and at P90, when *Mbp* mRNA levels have declined to stable levels and myelin is thought to be mature. At P14, a robust correlation between *Mbp* mRNA and *Mbp* protein was observed (Figure 2a,b) aligning with previous findings in brain (Popko et al., 1987; Shine et al., 1992). This observation underscores the pivotal role of transcriptional regulation in governing MBP protein levels in young mice. In contrast, at P90, lines with *Mbp* mRNA levels equal to or above 64% of WT (M3KOKI, M3(225)KO, M5KO, and M3KO) accumulated WT MBP protein levels. In the more severely affected M3M5KO and M1EM3M5KO lines (*Mbp* mRNA levels respectively 38% and 25%), MBP levels also improved but did not reach WT levels (Figure 2a–d). Notably, the level of CNPase, an oligodendrocyte protein not prominent in compact myelin (Trapp et al., 1988), was similar in all lines at both P14 and P90 (Figure 2a). For IMS, samples obtained at P90, and the intermediate age of P30, showed a trend similar to that revealed by WB. Thus, as mice mature, the close correlation between *Mbp* mRNA and protein levels is no longer maintained, minimizing the role of transcriptional regulation in governing MBP protein levels.

### 3.3 White matter volume correlates with *Mbp* mRNA levels

**FIGURE 3:**
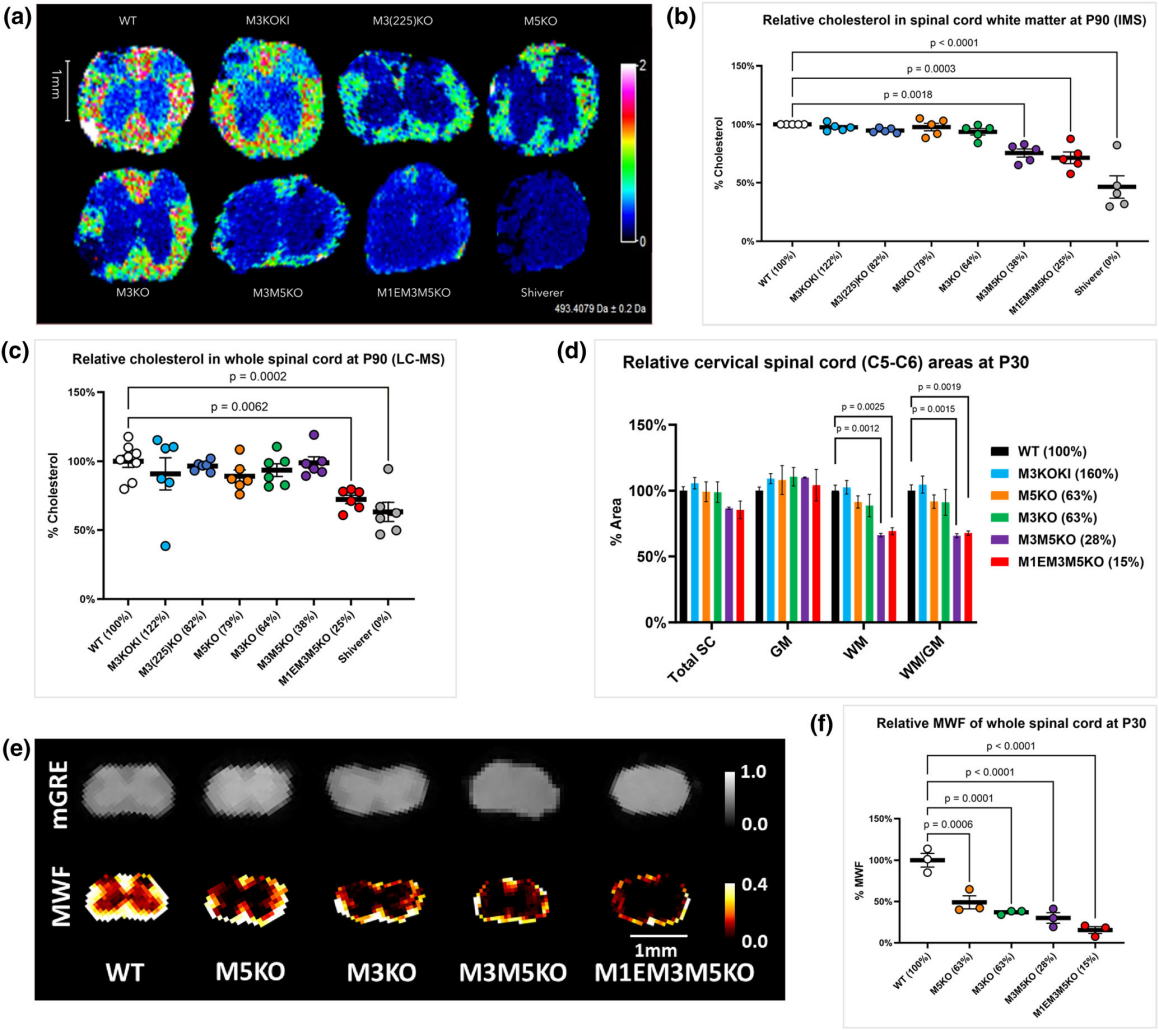
Multiple effects of reduced *Myelin basic protein* (*Mbp*) mRNA. (a) Cholesterol distribution and signal intensity in P90 cervical spinal cord measured by imaging mass spectrometry (IMS). (b) Relative cholesterol levels in cervical spinal cord white mater at P90, IMS (*n* = 5). (c) Relative cholesterol in whole cervical spinal cord at P90, liquid chromatography‐mass spectrometry (LC–MS) (*n* = 6–8). Cholesterol levels correlate with MBP protein levels at P90. (d) Relative cross‐sectional areas of cervical spinal cord (C5–C6) at P30 (*n* = 3–4). (e) myelin water fraction (MWF) and mGRE signals at P30 measured by MRI. (f) Relative MWF from combined GM and WM cervical spinal cord (C2–C7) at P30 (*n* = 3). White matter volume correlates with *Mbp* mRNA levels at P30. Relative mRNA values at P30 and P90 are indicated as a percentage of WT. All data are presented as mean, and error bars indicate SEM. *p*‐values are calculated using one‐way ANOVA and Dunnett's multiple comparisons test in (b,c,f), and multiple unpaired *t*‐tests in (d).

The membrane comprising myelin sheaths is atypical as 46% of its lipid content is cholesterol (Chrast et al., 2011; Norton & Poduslo, 1973). Therefore, as a potential surrogate of relative myelin content, we established the relative levels of cholesterol accumulation in the cervical spinal cord of P90 enhancer‐edited mice by both IMS and Liquid Chromatography‐Mass Spectrometry (LC–MS). In mice with *Mbp* mRNA levels 64% of WT or higher, cholesterol levels in spinal cord white matter were normal, but in those with lower *Mbp* mRNA levels (M3M5KO and M1EM3M5KO), cholesterol was significantly reduced. To estimate the cholesterol contribution from membrane other than that from compact myelin, we measured cholesterol in the dysmyelinated spinal cord of shiverer mice and found it reduced by 59% (IMS) and 37% (LC–MS) (Figure 3a–c). The greater reduction in the IMS signal is attributable to its origin exclusively from white matter domains, whereas LC–MS measurements were obtained from spinal cord homogenates containing both white and gray matter.

To evaluate potential effects of MBP reduction on the relationship between white and gray matter volumes in spinal cord, we evaluated their relative areas in cross‐sections of C5‐C6 spinal cord at P30. Consistent with the limited density of myelinated fibers in gray matter, *Mbp* reduction had little effect on the cross‐sectional area of that domain. In contrast, white matter area trended lower in M3KO and M5KO and was significantly lower in M3M5KO and M1EM3M5KO mice demonstrating that major changes in spinal cord structure arise from MBP mediated hypomyelination (Figure 3d).

Myelin volumes in C2–C7 spinal cords at P30 also were inferred from MWF measurements obtained by MRI. MWFs computed from multi‐echo gradient echo MRI data closely correlated with *Mbp* mRNA and protein levels and were significantly reduced in all hypomyelinated lines including those with the mildest mRNA reductions (M3KO and M5KO) (Figure 3e,f). It is unknown whether the marked signal reductions in M3KO and M5KO samples reflect a greater capacity of MRI to recognize subtle changes in myelin volume or otherwise undetectable myelin anomalies. Collectively, these findings show that reduced levels of *Mbp* mRNA and protein have a stepwise effect on myelin volume at young ages.

### 3.4 Sheath thickness correlates more closely with *Mbp* mRNA levels in young versus old mice

**FIGURE 4:**
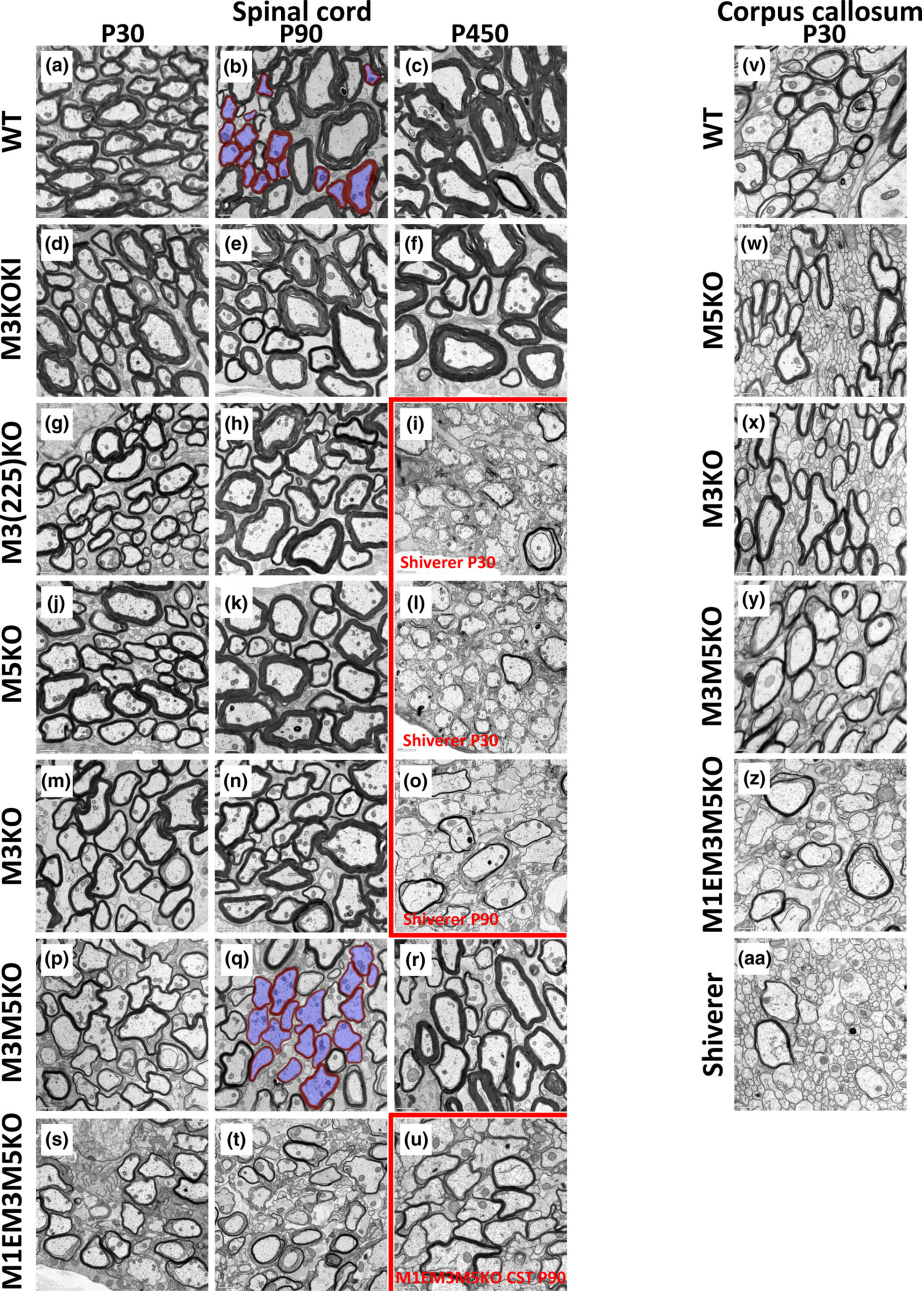
EM images from the cervical spinal cords and corpus callosum of enhancer‐edited mice. Gracile and cuneate tracts from: (a) P30 WT. (b) P90 WT. (c) P450 WT. (d) P30 M3KOKI. (e) P90 M3KOKI. (f) P450 M3KOKI. (g) P30 M3(225)KO. (h) P90 M3(225)KO. (i) P30 Shiverer. (j) P30 M5KO. (k) P90 M5KO. (l) P30 Shiverer. (m) P30 M3KO. (n) P90 M3KO. (o) P90 Shiverer. (p) P30 M3M5KO. (q) P90 M3M5KO. (r) P450 M3M5KO. (s) P30 M1EM3M5KO. (t) P90 M1EM3M5KO. (u) Corticospinal tract from P90 M1EM3M5KO. Myelin thickness correlates with Myelin Basic Protein (MBP) levels at both P30 and P90. Panels (b,q) show the segmentation of fibers meeting the selection criteria for inclusion in g‐ratio analysis (myelin in red and axons in blue). Scale bar (a–t) = 1 μm. Scale bar (u) = 0.5 μm. Cross sections from the body of the CC near midline at P30. (v) WT. (w) M5KO. (x) M3KO. (y) M3M5KO. (z) M1EM3M5KO. (aa) Shiverer. A step wise reduction in sheath thickness similar to that observed in spinal cord was apparent in the CC. Further, the myelin associated tubules prominent in spinal cord fibers from M1EM3M5KO and M3M5KO mice also were observed in the CC samples from mice of these same genotypes. Additionally, many small caliber axons remained unmyelinated in M1EM3M5KO mice at this age.

The observed myelin volume differences could arise from any combination of reduced sheath thickness or differences in the proportion of successfully myelinated axons. To determine how altered *Mbp* mRNA/protein accumulation affects myelin sheath thickness, EM images from spinal cord and CC were obtained from all lines of enhancer‐edited mice. Visual inspection of P30 samples revealed reduced myelin thickness closely correlating with *Mbp* mRNA level in both the GC and CC (Figure 4a‐aa). However, in lines with the smallest *Mbp* mRNA reductions (M3KO, M5KO and M3(225)KO), myelin thickness increased by P90. No improvement between P30 and P90 was obvious in M3M5KO or M1EM3M5KO mice but, obviously thicker myelin was present in fibers of M3M5KO mice at P450.

To investigate the axon‐myelin relationships in a more quantitative manner we compared g‐ratios (axon diameter/fiber diameter). Mice of all enhancer‐edited genotypes were investigated at P30 and P90 and to evaluate potential age‐related changes (Peters, 2002; Peters et al., 2000; Peters et al., 2001), WT, M3KOKI, and M3M5KO mice also were evaluated at P450.

**FIGURE 5:**
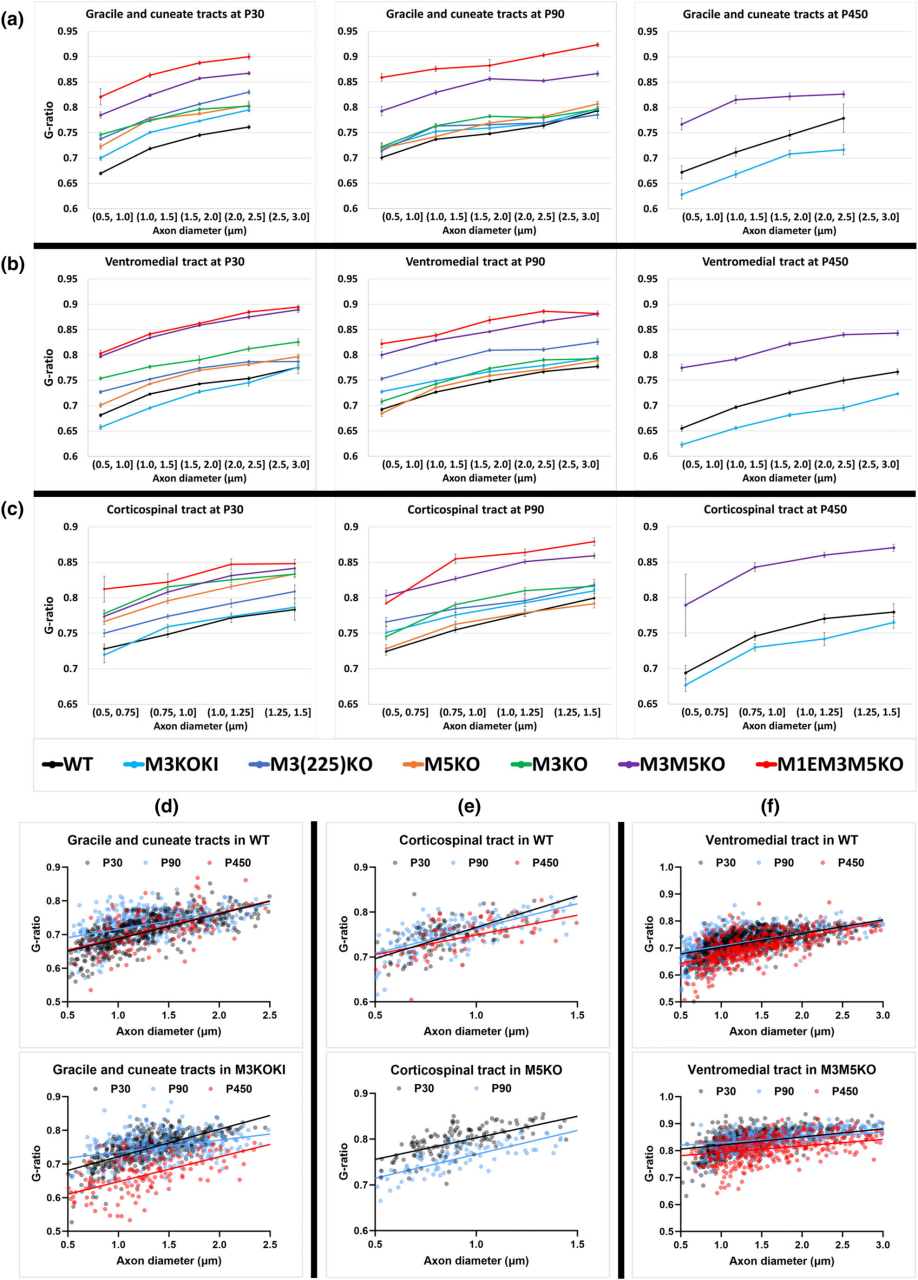
Spinal cord g‐ratio in sensory, motor and mixed tracts from enhancer‐edited mice at different ages. (a) Gracile and cuneate tracts at P30, P90, and P450. (b) Ventromedial (VM) tract at P30, P90, and P450. (c) Corticospinal tract at P30, P90, and P450. Each point represents mean g‐ratio per bin, and error bars indicate SEM (*n* = 1–2) (bin size = 2–495 fibers). Selected examples of age‐related changes in: (d) Gracile and cuneate tracts in WT and M3KOKI, (e) Corticospinal tract in WT and M5KO, (f) VM tract in WT and M3M5KO (*n* = 1–2). Myelin thickness in hypomyelinated lines improves or normalizes with age at a rate dependent on *Myelin basic protein* (*Mbp*) mRNA levels. At P450, M3KOKI line shows hypermyelination in all tracts.

To investigate possible heterogeneity between sensory and motor fibers, the g‐ratios in sensory GC tracts, motor CST, and mixed sensory and motor VM domains were examined. Only fibers free of split myelin and demonstrating close axon association over >90% of the axon circumference were selected for analysis; a circumstance limiting this analysis to axons with diameters ranging from 0.5–3.0 μm (Figure 4a–u). As replicate samples obtained from M3KO and M3KOKI lines yielded similar g‐ratio results, this survey was carried out using samples from one mouse of each genotype and age (Supplementary Figure S2). To represent the g‐ratio relationships in the three tracts and three ages examined, results are displayed as bin averages (Figure 5a–c). Also, to display age‐related g‐ratio changes more clearly, scatter plots of the original data were generated from the selected lines (Figure 5d–f).

At P30, the most severely affected M1EM3M5KO line (15% *Mbp* mRNA) maintained the thinnest sheaths. M3M5KO mice (28% *Mbp* mRNA), had modestly thicker sheaths while the more mildly affected M3KO, M5KO and M3(225)KO lines (58%–63% *Mbp* mRNA) maintained sheaths that were only slightly thinner than normal. Mice in the over‐expressing M3KOKI line (160% *Mbp* mRNA) elaborated sheaths that were normal in CST, modestly thicker in VM and slightly thinner in GC (Figure 5a–c). Consequently, the g‐ratios in all six enhancer‐edited lines reflected their relative *Mbp* mRNA levels.

*Mbp* mRNA accumulation declines in WT and enhancer‐edited mice between P30 and P90 following the normal developmental program but at different line‐specific levels (Figure 1a). During this period, no obvious changes in myelin thickness occurred in WT mice, but in M3KO, M5KO and M3(225)KO mice sheath thickness improved to approximate the WT value (Figure 5a–f). In the more severely deficient M3M5KO and M1EM3M5KO lines, no similar improvement was obvious at P90. However, while WT g‐ratio remained largely unchanged up to P450, modest improvement was apparent in the M3M5KO line. These combined observations demonstrate the existence of an optimal g‐ratio that is maintained in WT and suggest that the adult recovery response in hypomyelinated lines is rate‐limited by *Mbp* mRNA availability (Figure 5a–c,f). Curiously, in M3KOKI mice, *Mbp* mRNA levels were normal at advanced ages (P290 and P460) but axons of all calibers in all tracts were dramatically hypermyelinated at P450 (Figure 5a–d); an observation precluding a direct relationship between *Mbp* mRNA levels and myelin thickness at this advanced age.

Myelin proteins define interlamellar spacing and therefore, overall sheath thickness is the combined result of lamellae number and spacing. To determine if interlamellar spacing is perturbed when MBP is reduced, we evaluated the markedly thinner sheaths of M3M5KO mice. At P30, spacing was indistinguishable from normal (10.78 nm, fiber # = 57 vs WT 10.87 nm, fiber # = 23, *p*‐value = 0.5130) supporting the view that the reductions in myelin thickness reflect only lamellar number.

Axon caliber is reported to be affected by lack of normal myelin (Shine et al., 1992). If hypomyelination consequent upon *Mbp* deficiency had a similar effect, it would complicate interpretation of g‐ratio calculations that presume equal axon diameter distributions in all samples. The calibers of all complete axon profiles in all available images (20–30) from the GC tracts from all enhancer‐edited lines at P30 were compared to WT. Axon diameters also were compared between P30 and P450 WT and P450 M3M5KO and M3KOKI mice (Supplementary Figure S3). A large overlap was observed demonstrating that axon diameters are largely unaffected by *Mbp* induced changes in myelin thickness in both young and old mice.

### 3.5 Sheath length is reduced only in mice with severely diminished *Mbp* mRNA levels

**FIGURE 6:**
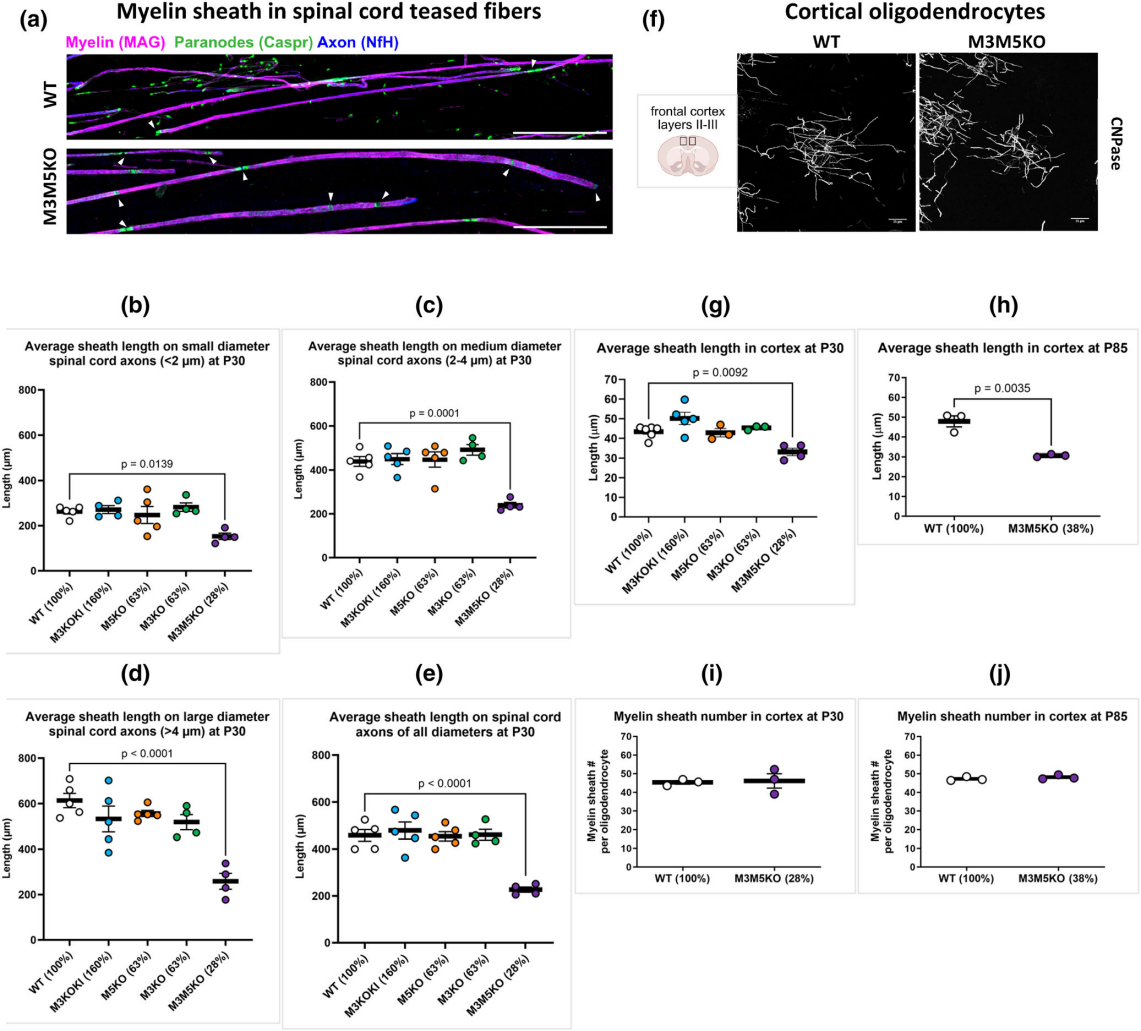
Effect of reduced *Myelin basic protein* (*Mbp*) mRNA on length and number of myelin sheaths elaborated by oligodendrocytes. (a), Teased spinal cord fibers. Arrows indicate ends of myelin sheaths as identified by Caspr (green). Scale bar = 100 μm. Average sheath length: (b) axons <2 μm, c, axons 2–4 μm, d axons >4 μm, e axons of all diameters. There is a significant decrease in mean myelin sheath length for M3M5KO mice (*n* = 4). There was no difference in mean sheath lengths in M3KO (*n* = 4), M5KO (*n* = 5) or M3KOKI bp (*n* = 5) mice compared to WT (*n* = 5). (f) Schematic of the cortical region analyzed and confocal micrographs of single oligodendrocytes. Scale bar = 25 μm. (g), Average sheath length in cortex at P30, and (h), at P85. Mean myelin sheath lengths per mouse shows reduced lengths only in M3M5KO mice at both P30 and P85. (i) Myelin sheath number per oligodendrocyte at P30, and (j) at P85. The number of myelin sheaths per individual oligodendrocyte is not affected in M3M5KO mice. Relative mRNA values at P30 and P90 are indicated as a percentage of WT. *p*‐values are calculated from one‐way ANOVA and Dunnett's multiple comparisons test. All data are presented as mean per mouse, and error bars indicate SEM (*n* = 3–6).

In addition to axon caliber and myelin thickness, spacing of nodes of Ranvier greatly affects action potential conduction rate (Cohen et al., 2020). Notably, myelin sheaths on large caliber axons are not only thicker but extend along axons for greater distances than those on small caliber axons (Hess & Young, 1949; Waxman & Sims, 1984). The mechanisms that determine sheath length are yet to be fully characterized, but compelling evidence suggests that nodal spacing can be prefigured on some axons (Vagionitis et al., 2022), while intrinsic oligodendrocyte properties and axon diameter control spacing on others (Bechler et al., 2015). To assess whether internodal distance is affected by reduced *Mbp* mRNA levels, we investigated teased fibers obtained from spinal cord white matter immunolabeled for markers of myelin, nodes, and paranodes (Figure 6a). Despite the reduced sheath thickness observed at P30 in M3KO and M5KO mice (63% *Mbp* mRNA), their sheath lengths were normal on fibers with large, medium, and small calibers. In contrast, the more severe reduction in sheath thickness observed in M3M5KO mice (28% *Mbp* mRNA), was associated with an approximately 50% reduction in sheath lengths on fibers of all calibers (Figure 6b–e, Supplementary Figure S4). Thus, internodal length is less sensitive to reduced MBP levels than sheath thickness. Further, reduced sheath lengths in M3M5KO mice were associated with abnormalities in nodal and paranodal labeling patterns consistent with overlapping paranodes and trailing paranodal loops (Supplementary Figure S5).

To investigate the length and number of myelin sheaths elaborated by individual oligodendrocytes, we turned to the sparsely myelinated frontal cortex where oligodendrocytes, along with their full complement of myelin sheaths, can be visualized (Figure 6f). Consistent with the observations on teased spinal cord fibers, the lengths of cortical sheaths were normal in M3KO and M5KO mice but reduced in the more affected M3M5KO line, at both P30 and P85 (Figure 6g,h). As cortical myelin sheaths terminate as heminodes, such shortening is uncomplicated by potential competition from adjacent sheaths. Further, the number of sheaths elaborated by each cortical oligodendrocyte was normal in all lines including M3M5KO (Figure 6i,j), an observation demonstrating that MBP, a late expressing myelin gene, does not play a role in establishing sheath number.

### 3.6 Capacity of oligodendrocytes to ensheath axons correlates with *Mbp* mRNA levels

**FIGURE 7:**
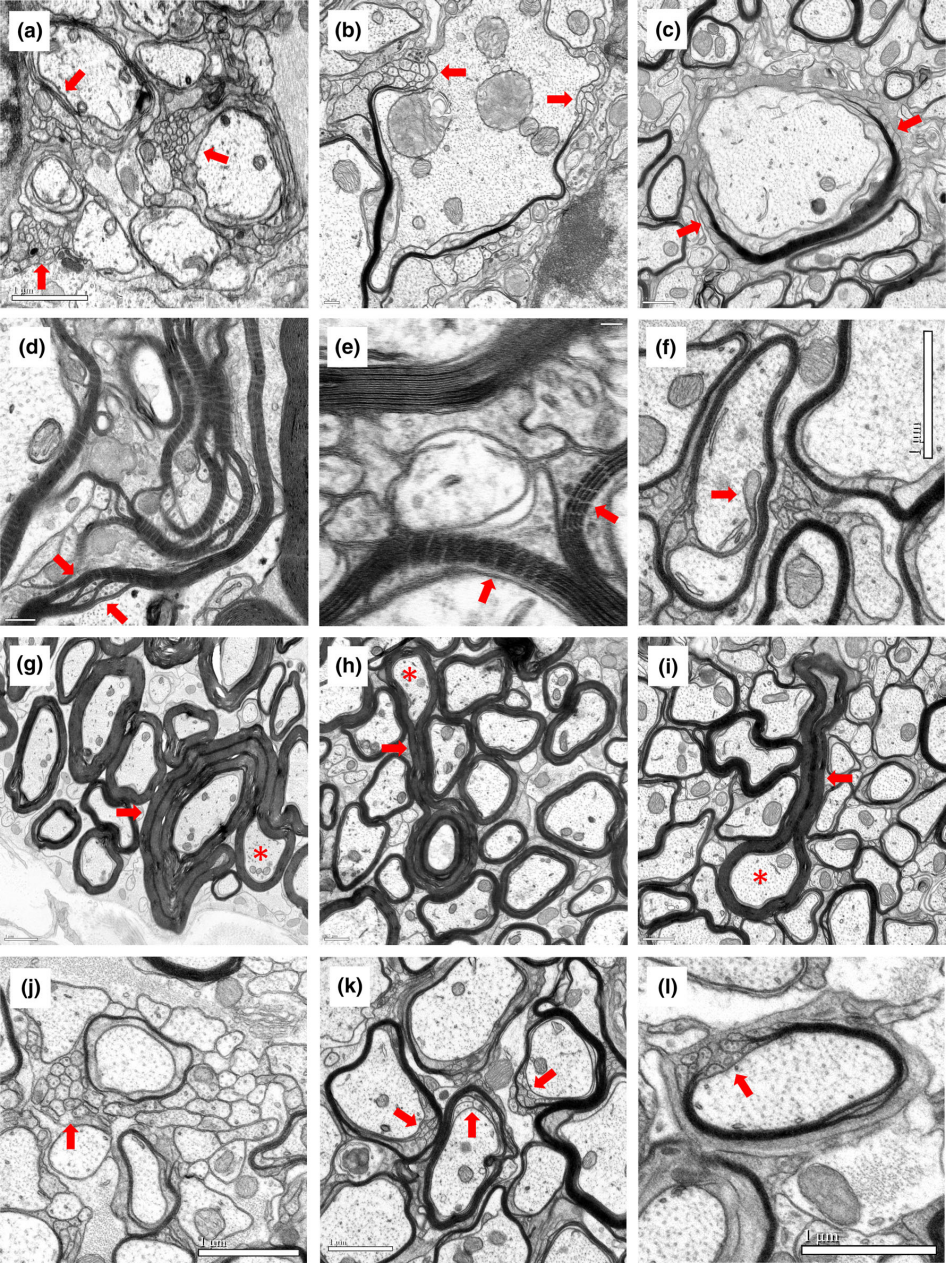
Abnormal spinal cord fiber profiles. (a) Clusters of oligodendrocyte processes in gracile and cuneate (GC) tract of P30 Shiverer. (b) Partially ensheathed axon with membrane tubules in ventromedial tract of P90 Shiverer. (c) Partially ensheathed axon with membrane tubules in corticospinal tracts (CST) tract of P450 M3M5KO. (d) Microtubule containing myelinic channels in GC tract of P450 M3M5KO. (e) Radial components crossing myelin sheath between inner and outer tongue in GC tract of P30 M5KO. (f) Oligodendrocyte process invading the axon in GC tract of P90 M1EM3M5KO. (g) Myelin outfolding in GC tract of P450 WT. (h) Myelin outfolding in GC tract of P30 M5KO. (i) Myelin outfolding in CST tract of P450 M3M5KO. (j) Clusters of membrane tubules in GC tract of P90 M1EM3M5KO. (k) Membrane tubules in GC tract of P90 M1EM3M5KO. (l) Membrane tubules in GC tract of P90 M1EM3M5KO. Red arrows are pointing to the features. Axons with outfoldings are designated with red asterisks. Scale bar = (a,f,g,j,k,l): 1 μm, (c,h,i): 0.5 μm, (b,d): 0.2 μm, (e): 50 nm.

Like lamellipodia in other cell types, the forward progress of the inner tongues of myelin sheaths requires actin turnover and a role for MBP has been implicated in such actin dynamics (Nawaz et al., 2015; Snaidero et al., 2014; Zuchero et al., 2015). This suggests that low MBP levels could compromise the forward movement of the inner tongue, thus attenuating the capacity of oligodendrocyte membrane to circumnavigate axons resulting in the different g‐ratios observed in the enhancer‐edited lines. Consistent with that model, many axons in *Mbp* null shiverer mice lack spirally wrapped oligodendrocyte membrane altogether and, when membrane wrapping is present, only a few uncompacted layers are observed (Figures 4i, l, o and 7a,b). To determine if reduced MBP levels also compromise the ability of oligodendrocytes to initiate a myelination program, we searched for the presence of dysmyelinated axons in the different enhancer‐edited lines. M1EM3M5KO mice accumulated only 15% of the WT *Mbp* mRNA level at P30 and revealed a sizable subpopulation of entirely dysmyelinated axons across a wide range of calibers (Figure 4s–u). Other axons were associated with sheets of compact myelin that failed to cover the entire axon circumference (Figure 7c) while the remainder were surrounded by uninterrupted, albeit exceptionally thin, compact myelin. However, at mRNA levels 28% or above, all caliber‐appropriate spinal cord axons appeared to be myelinated (Figure 4j, k, m, n, p, r). Also, in the CC of P30 M1EM3M5KO mice, many caliber‐appropriate axons remained unmyelinated (Figure 4z).

### 3.7 Striking myelin sheath abnormalities correlate with *Mbp* mRNA levels

In the developing CNS of young WT mice, myelin sheaths typically display myelinic channels and large microtubule‐containing inner tongues but with maturity, these features become less prominent (Snaidero & Simons, 2014). In contrast, these features persisted in the markedly hypomyelinated M3M5KO and M1EM3M5KO lines (Figure 7d). Notable in all lines, prominent clusters of radial components traversing myelin sheaths frequently were located where inner and outer oligodendrocyte tongues aligned (Figure 7e). Rarely, finger‐like oligodendrocyte processes protruded into the axon (Figure 7f).

Myelin outfoldings meander between, and often surround, adjacent myelinated axons and are frequently observed in multiple diseases states as well as in normal animals at both preweaning and advanced ages (Erwig et al., 2019; Peters, 2002; Peters et al., 2000; Peters et al., 2001; Snaidero et al., 2014; Sturrock, 1976). In all enhancer‐edited mice and WT, myelin outfoldings were rare at P30 and P90 but more common at P450 (Figure 7g,i). Despite the attenuated capacity of M3M5KO mice to elaborate sheaths with normal thickness and length, many fibers had prominent outfoldings of compact myelin with a thickness similar to that part of the sheath surrounding the axon (Figure 7i). This feature limits the nature of the mechanism underlying their appearance with one attractive possibility being shortening of preexisting sheaths without a change in the initial sheath volume. Note that outfoldings arise from sheaths associated with axons across a wide range of calibers.

In both the spinal cord and CC of M1EM3M5KO mice, a large population of fibers with prominent clusters of membrane tubules were observed (75% at P30 and 70% at P90 of GC fibers) (Figure 7j–l). In both GC and CC, such profiles were encountered less frequently in M3M5KO mice and not all in M3KO and M5KO mice. Notably, they were less frequent in VM and CST, suggesting potential inter‐tract heterogeneity.

### 3.8 Graded changes in myelin elicit stepwise responses in glial cell density

**FIGURE 8:**
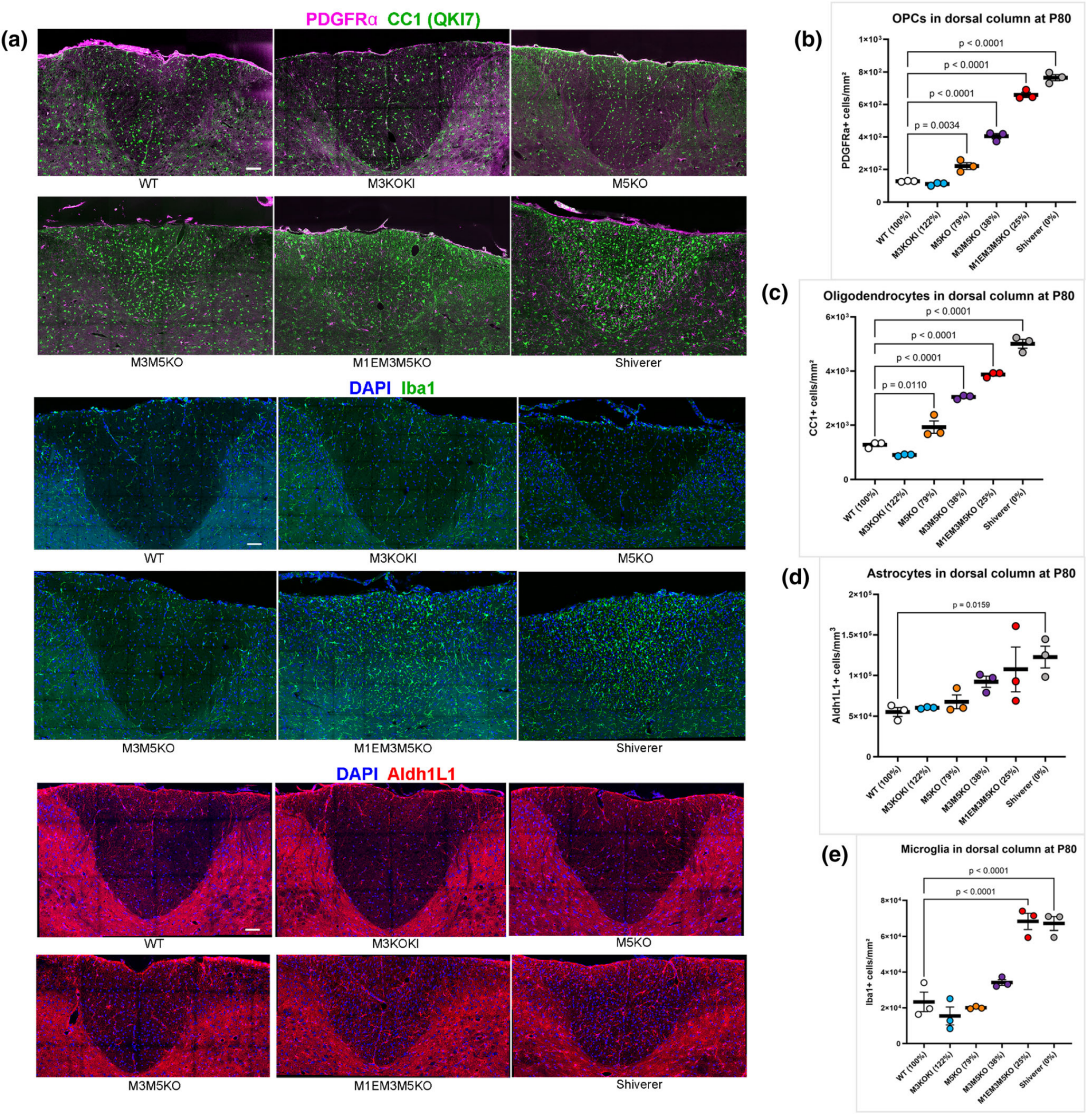
Density of glial cells in dorsal column at P80. (a) Immunohistochemical staining of oligodendrocytes (green) and OPCs (purple), microglia (green) and astrocytes (red) in dorsal cervical spinal cord. Scale bar = 50 μm. (b) OPC density. (c) oligodendrocyte density. (d) Astrocyte density. (e) Microglia density. Density of OPCs, oligodendrocytes and astrocytes correlates negatively with *Myelin basic protein* (*Mbp*) mRNA levels. Increased density and morphological changes of microglia occur only in M1EM3M5KO and shiverer mice. Relative mRNA values are indicated as a percentage of WT at P90. *p*‐values are calculated using one‐way ANOVA and Dunnett's multiple comparisons test. All data are presented as mean, and error bars indicate SEM (*n* = 3).

It is well documented that different glial lineages establish close physical interactions with myelinated fibers extending to synaptic contacts (Rasband & Peles, 2016). As previously reported (Bu et al., 2004), we show that the density of oligodendrocyte progenitor cells (OPCs) and post‐mitotic oligodendrocytes in the dysmyelinated dorsal columns of P80 shiverer mice is respectively, 6 and 3.9 times higher than WT. To determine how this response relates to the degree of hypomyelination, their density was evaluated across the enhancer‐edited panel and a closely correlating inverse relationship between density and hypomyelination status was observed. Thus, the mechanism regulating their density is sensitive to even subtle myelin changes (Figure 8a–c). To determine if other glial lineages mount a similar response, the densities of dorsal column astrocytes and microglia were evaluated using Aldh1L1 and Iba1 as respective lineage markers (Figure 8a). Like the oligodendrocyte lineage, astrocyte density also correlated with the extent of hypomyelination (Figure 8d). In contrast, the microglia population remained normal in modestly affected lines, but increased density and morphological changes were observed in M1EM3M5KO and shiverer mice suggesting that a significant inflammatory response occurs only with severe hypo/dysmyelination (Figure 8e).

### 3.9 Only mice with the most severely reduced *Mbp* mRNA level exhibit abnormal behavior and shortened lifespan

The enhancer‐edited mice were established through backcrosses to C57Bl/6 and are maintained as homozygotes. Mice in five of the six lines were indistinguishable from WT by in‐cage behavior, reproductive capacity, life span, and body weights thus minimizing the possibility of secondary effects on myelin phenotypes (Supplementary Figure S6). However, M1EM3M5KO mice, that accumulated the lowest mRNA level, demonstrated a mild shiverer‐like phenotype initiating around P18 along with frequent seizures after maturity. While their body weights were lower at older ages, and their lifespan reduced to approximately 9 months, their reproductive capacity was not compromised. F1 offspring from a cross between M3M5KO and M1EM3M5KO are predicted to accumulate an intermediate *Mbp* mRNA level and had a normal lifespan and demonstrated subtle shivering and rare seizures only when young.

## 4 DISCUSSION

Our observations on young enhancer‐edited mice demonstrated a close correlation between myelin sheath thickness and *Mbp* mRNA and protein accumulation. We show here that such correlations extend to myelin volume measurements including the area of white matter tracts, cholesterol content, and MWF. We also show that severely reduced *Mbp* mRNA levels diminished the capacity to initiate sheath elaboration and the ability to extend sheathes longitudinally. Also correlating with *Mbp* mRNA levels was the presence of prominent myelin‐associated membrane tubule clusters. Unexpectedly, we also show that initially hypomyelinated sheaths improve or normalize with age, further defining the dynamic nature of myelin in the mature CNS. Such plasticity is played out over a protracted period when stable and low levels of *Mbp* mRNA are maintained, with the actual rate of improvement correlating with line specific *Mbp* mRNA levels. While, these observations imply engagement of a post‐transcriptional mechanism, defining the precise roles played by transcriptional and post‐transcriptional mechanisms is precluded due to the long half‐life of MBP protein sequestered in myelin sheaths (Meschkat et al., 2022) and the unknown stability of RNP‐associated *Mbp* mRNA. It also is unclear if the *Mbp* mRNA level existing at the time of analysis, or earlier during the short period required to elaborate a sheath (Czopka et al.,^2013^), has the most influence on establishing sheath dimensions.

The conclusions drawn from this investigation contrast with those of the earlier transgene‐based investigations in which a linear relationship between *Mbp* mRNA and MBP protein was proposed to persist from early development through maturity (Shine et al., 1992). That relationship was inferred from *Mbp* mRNA levels at a young age (P18) and MBP protein measured at maturity (P60). However, the sequence of the *Mbp* transgene used in those studies is now known to lack both the M3 and M5 oligodendrocyte enhancers that are major contributors to *Mbp* expression. Consequently, the actual level of *Mbp* mRNA that arose in the P60 brain from the randomly inserted transgene on a mixed genetic background is unknown. Therefore, a direct comparison with the present results cannot be made leaving open the possibility that the earlier data could be accommodated by a model in which post transcriptional mechanisms gain importance with maturity.

The thickness and length of myelin sheaths corresponds to the caliber of the ensheathed axon (Hess & Young, 1949; Waxman & Sims, 1984) implying that control of myelin sheath dimensions originates at the level of individual internodes. As revealed by the g‐ratio analysis of enhancer‐edited mice, and shown previously (Shine et al., 1992), altered *Mbp* mRNA levels affect sheath thickness on axons of all calibers (Figure 5a‐c). As sheaths with vastly different volumes exist on small vs large caliber axons, it remains an enigma how reduced *Mbp* transcription could affect sheath dimensions on all axons in an apparently equivalent manner. As *Mbp* mRNA is transported to the myelin sheath prior to translation (Barbarese et al., 1995; Carson et al., 2001), two general mechanisms are suggested. Either different concentrations of *Mbp* mRNA are recruited to individual sheaths or uniformly distributed *Mbp* mRNA is translated with different efficiencies depending upon axon caliber. Supporting the potential importance of mRNA transport, numerous microtubule‐disrupting mutants result in hypomyelination (Duncan et al., 2017; Fu et al., 2019; Herbert et al., 2017; Lyons et al., 2009; Seiberlich et al., 2015). Regardless, both models depend upon signals to the myelin sheath that must originate locally from the ensheathed axon.

Our observations arise directly from the experimentally imposed changes in *Mbp* mRNA accumulation. While the minimum concentration of MBP required to compact spirally wrapped oligodendrocyte membrane into myelin sheaths remains known, the correlation observed here between sheath dimensions and *Mbp* mRNA levels supports the view that all compact myelin, regardless of its normal or hypomyelinated state, has an equivalent concentration of MBP. A similar stoichiometry was observed for myelin physically isolated from *mld/mld* mice (Roch et al., 1987).

In all mouse lines in which the M3 enhancer was edited or deleted, *Golli* mRNA accumulation was abnormal (Bagheri et al., 2020; Dib et al., 2011). As the total lack of *Golli* disrupts myelin formation on fibers serving the optic system (Jacobs et al., 2005), its widespread disruption in the M3‐deleted lines might contribute to the observed myelin phenotypes thus complicating interpretation of the present results. However, M3KO and M5KO mice accumulated similar levels of *Mbp* mRNA, and achieved equivalent myelin phenotypes despite the major reduction in *Golli* mRNA accumulation that occured only in M3KO mice; an observation that rules out any contribution of the *Golli* mRNA reduction to the spinal cord myelin phenotypes reported here. Nonetheless, *Golli* overexpression in M3KO389bpKI mice (145% of WT) could potentially contribute to the hypermyelination observed in such mice at P450, an age when *Mbp* mRNA has returned to WT levels.

Our observations on myelin sheath thickness in the enhancer‐edited mice are accommodated by the current model of sheath formation in which the lamellapodia‐like inner tongue circumnavigates the axon to deposit spirally wrapped layers of oligodendrocyte plasma membrane (Snaidero & Simons, 2014). Traditionally, MBP was thought to compact and stabilize myelin but more recently, a capacity to modulate cytoskeleton dynamics also was recognized. Consequently, in its absence or reduced concentration, inner tongue function could be compromised (Zuchero et al., 2015). Consistent with MBP playing a critical role in lamellipodia function, massive clusters of small tubular membrane profiles are interspersed between the dysmyelinated axons of *Mbp* null shiverer mice. Similar clusters were a notable feature of many fibers in the most severely hypomyelinated lines (M3M5KO and M1EM3M5KO) but were not observed in M3KO and M5KO mice (63% of WT). In hypomyelinated fibers, such structures typically extend directly from regions of compact myelin lamellae providing further insight into their origin.

In addition to the effects of altered *Mbp* mRNA on myelin formation, the densities of OPCs, oligodendrocytes, astrocytes, and microglia increased relative to hypomyelination severity. These secondary responses could be directed by the hypomyelinated fibers themselves or mediated through interactions among the different glial lineages, as seen with the response of OPCs to activated microglia (Hughes & Appel, 2020; McNamara et al., 2023; Santos & Fields, 2021; Sherafat et al., 2021; Traiffort et al., 2020). As revealed by the stepwise response across the enhancer‐edited lines, the underlying mechanism affecting OPCs, oligodendrocytes and astrocytes is exquisitely sensitive to very small differences in MBP levels and/or myelin dimensions.

Marked heterogeneity has emerged among different oligodendrocyte populations (Bechler et al., 2015; Gargareta et al., 2022; Marques et al., 2016; Seeker et al., 2023; Spassky et al., 1998) thus putting the generalizability of observations on spinal cord to other CNS domains in doubt. However, in the distinctly different spinal cord and cortical domains, altered *Mbp* mRNA levels affected myelin sheath lengths equivalently and alterations in sheath thickness were largely equivalent in motor and sensory tracts in the spinal cord. Nonetheless, inter‐tract changes in g‐ratios were observed in those lines in which the M3 enhancer was edited to reduce its complement of conserved transcription factor binding sites. As similar inter‐tract heterogeneity was realized by reporter constructs similarly regulated by M3 sub‐sequences, such differences might reflect heterogeneity in the transcription factor repertoire expressed by oligodendrocytes in different tracts. The exceptionally high *Golli* mRNA accumulation observed in the optic nerve of M3KOKI mice is one striking example of such domain‐specific heterogeneity.

Mice in the enhancer‐edited lines are long‐lived with typical reproductive capacity. In all but one line, no remarkable in‐cage behaviors nor obvious progressive pathology was observed. These readily accessible mice and their initial characterization, as provided here, should facilitate investigations on diverse aspects of the myelination program (Liu et al., 2022) that operates over the course of the mouse lifespan.

## AUTHOR CONTRIBUTIONS

H. B., H. F., A. H., C. J., A. P.: Derivation of enhancer‐edited mice, mRNA analysis, myelin ultrastructure analysis, funding and writing of manuscript; E. C.: Study design and editing of manuscript; L. O., C. G., A. K.: Study design and segmentation analysis; A. C., J. C‐A.: Axon‐myelin segmentation; A. Y., G. M. V., M. S., A. J, M. B.: Sheath length and number analysis, study design, editing manuscript; R. S. P., P. C.: IMS, study design and editing of manuscript; L. C., D. V.: LC–MS; E. W., O. G., A. N.: Glial density analysis, study design, editing of manuscript; A. B‐B., T. O.: Study design and glia evaluation; V. G., M. T., H. L., D. R.: MRI acquisition, MRI analysis and editing of manuscript; J. Z., K. S.: Western blots, study design and funding.

## FUNDING INFORMATION

K. S. and A. P.: Canadian Institutes of Health Research. A. K.: Natural Sciences and Engineering Research Council of Canada (NSERC) and McGill Initiative in Computational Medicine (MiCM). M. B.: Esther A. & Joseph Klingenstein Fund and the Simons Foundation. D. V.: NSERC DG RGPIN‐2019‐06973 and FRQS Chercheur‐Boursier 312947. A. N.: NIH R01NS073425, R01NS116182. NIH Instrumentation Grant S10 OD016435. H. L.: Brain Canada Research Grant and the Multiple Sclerosis Society of Canada (MSSC) EndMS Postdoctoral Fellowship. V. G.: EndMS doctoral award and FRQS doctoral award. D. R.: National Sciences and Engineering Research Council of Canada (NSERC) Discovery Grant (RGPIN‐2018‐05047) and Canada First Research Excellence Fund (CFREF), Healthy Brains for Healthy Lives Innovative Ideas Project Grant.

## CONFLICT OF INTEREST STATEMENT

The authors declare no conflicts of interest.

## Supporting information


**Data S1:** Supporting Information.

## Data Availability

The data that support the findings of this study are available from the corresponding author upon reasonable request.
