# Supplementary material for: Myelin basic protein mRNA levels affect myelin sheath dimensions, architecture, plasticity, and density of resident glial cells
Source: Glia. 2024 Jul 18;72(10):1893–914. doi: 10.1002/glia.24589 (PMC11426340; doi:10.1002/glia.24589)
Supplement: Supplementary file 1 — Data S1: Supporting Information. [file GLIA-72-1893-s001.docx]

**SUPPLEMENTARY FIGURES AND TABLES**


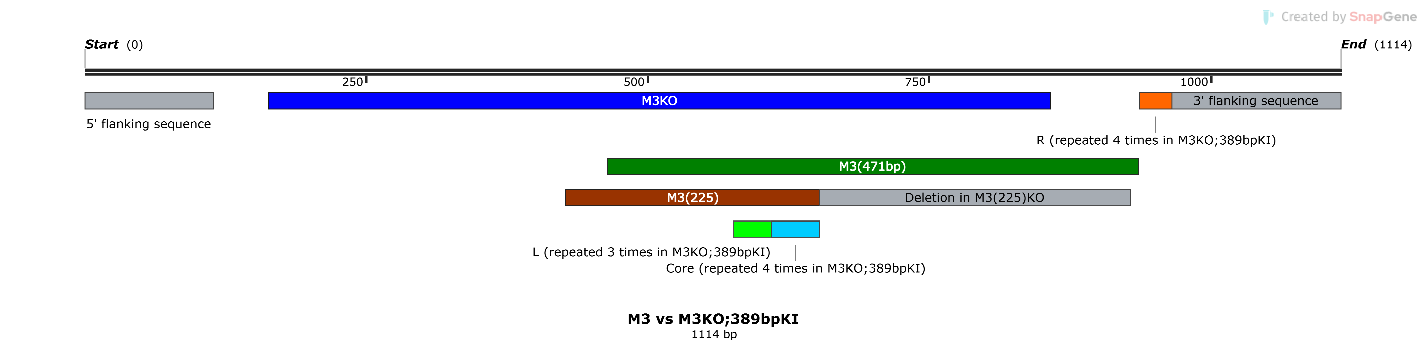


**B**

**A**


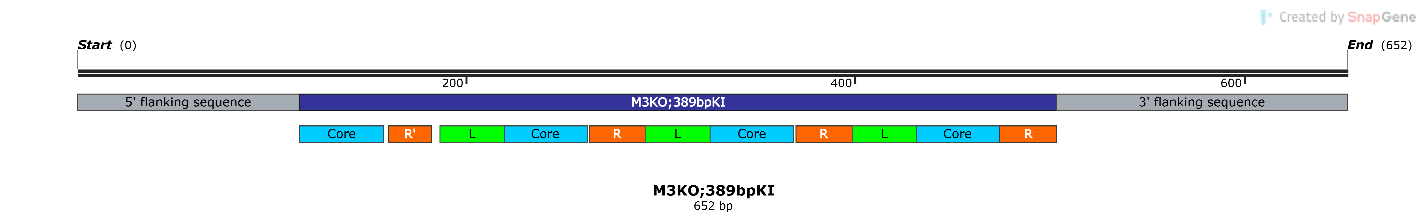


**C**

GGACACAAAGCCCAACTGTTGTAAAAATATTAGTATTCAGATGCCGCACTCAGTCTTGCCCTTTCCTTTGGGCCTCTCTGTATCTCACAAATAACTGTATTCAAAGGACACAAAGCCCAACTGTTGTAAAAATATTAGTATTCAGATGCCGCCCACTCAGTCTTGCCCTTTCCTTAGGCCTCTCTGTATCTCACAAATAACTGTATTCAAAGGACACAAAGCCCAACTGTTGTAAAAATATTAGTATTCAGATGCCGCCCACTCAGTCTTGCCCTTTCCTTAGGCCTCTCTGTATCTCACAAATAACTGTATTCAAAGGACACAAAGCCCAACTGTTGTAAAAATATTAGTATTCAGATGCGCCCACTCAGTCTTGCCCTTTCCTTAGG

**Supplementary figure 1. Schematic and sequence of M3KO;389bpKI allele. A,** Representation of the highly conserved M3(471bp) sequence, the M3KO and M3(225)KO alleles, and the M3 sub-sequence multimerized in the M3KO;389bpKI allele (abbreviated throughout this communication as M3KOKI). **B,** M3KOKI allele. **C,** M3KOKI sequence.


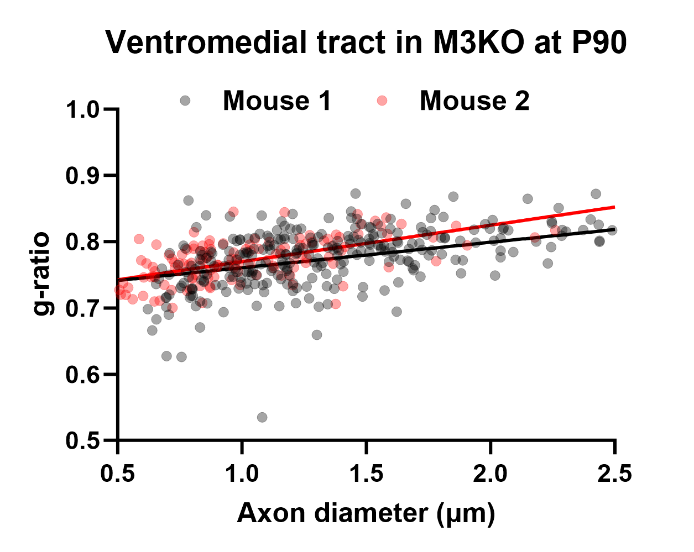


**A**

**B**


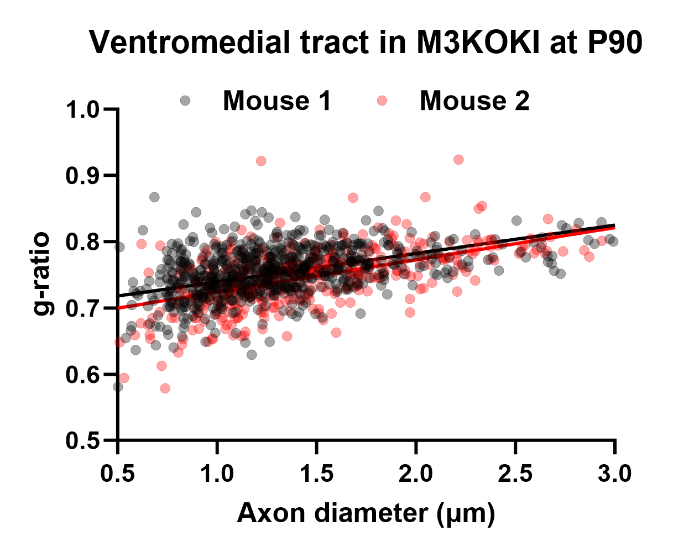


**C**


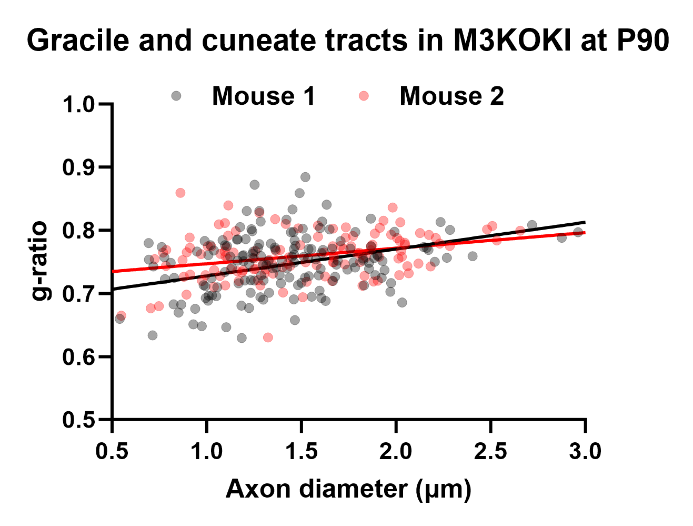


**Supplementary figure 2. G-ratio comparison between replicate mice at 90.**

**
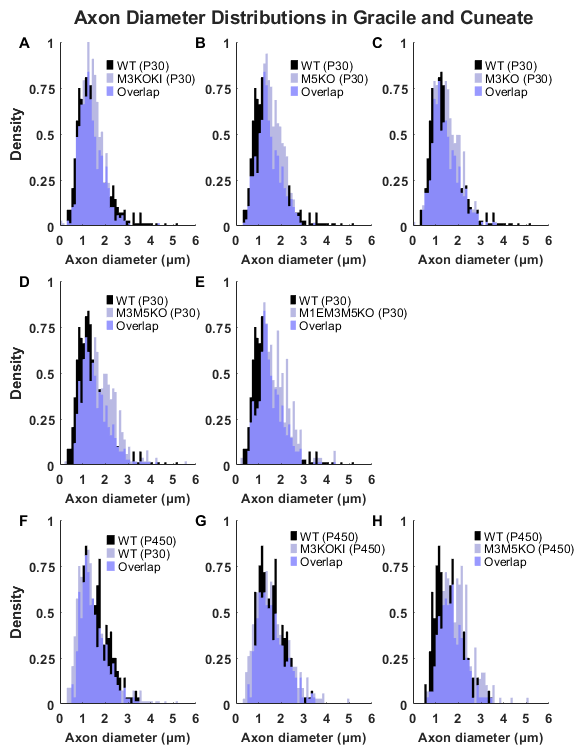
**

**Supplementary figure 3. Axon diameter distributions in gracile and cuneate tracts of enhancer-edited mice.** Axon diameters in non-overlapping images from gracile and cuneate tracts were measured in cross sections of all enhancer-edited and control mice at P30 and P450. All comparisons revealed an extensive overlap. At P30: WT (n = 680, Mean = 1.44µm); M3KO (n = 444, Mean = 1.49µm); M5KO (n = 555, Mean = 1.53µm); M3M5KO (n = 680, Mean = 1.44µm); M1EM3M5KO (n = 260, Mean = 167µm); M3KOKI (n = 682, Mean = 1.4µm). At P450: WT (n = 279, Mean = 1.44µm); M3M5KO (n = 293, Mean = 1.9µm); M3KOKI (n = 346, Mean = 1.61µm).


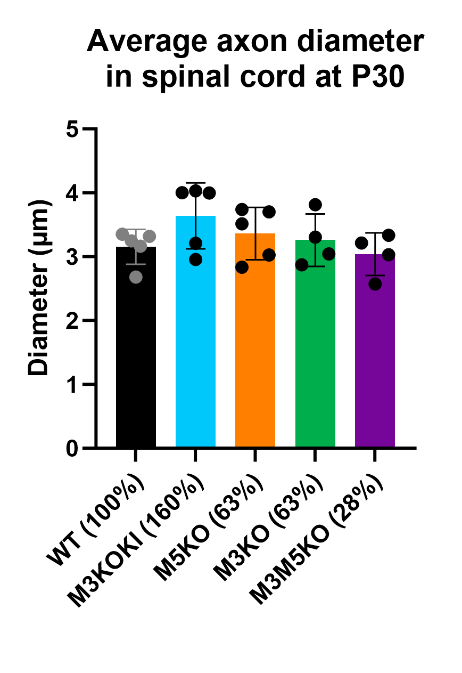
**
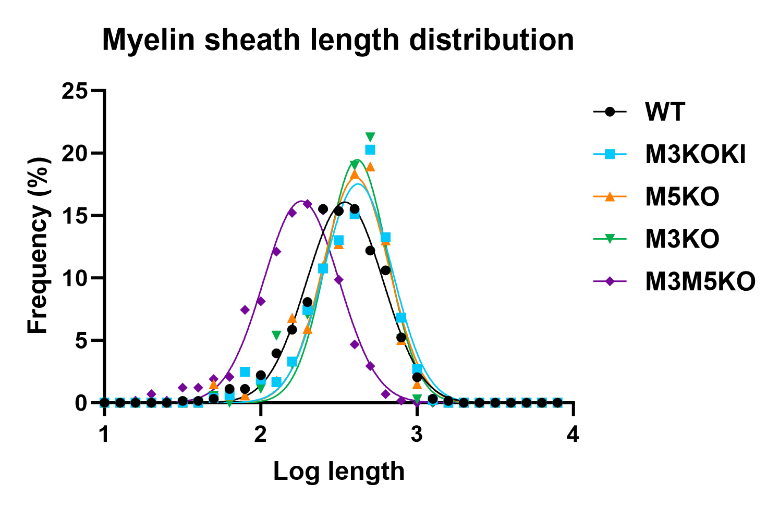
**

**B**

**A**

**Supplementary figure 4: Comparison of sheath length and axon size distribution in spinal cord teased fibers of P30 mice. A,** Frequency distribution of the log sheath lengths for each genotype. **B,** Teased spinal cord axon diameters were comparable between genotypes. Each dot is the mean axon diameter per mouse, assessed for axons where internode lengths were measured in Figure 10. (one-way ANOVA with Tukey’s post hoc analysis conducted)

**Supplementary figure 5. Altered paranodal features in P35 M3M5KO mice compared to wild type.** Overlapping paranodes were less distinct or shortened nodes of Ranvier in between Caspr doublets. Partial paranodes were single Caspr bands associated with Na_v_ channel staining and the absence of MAG.


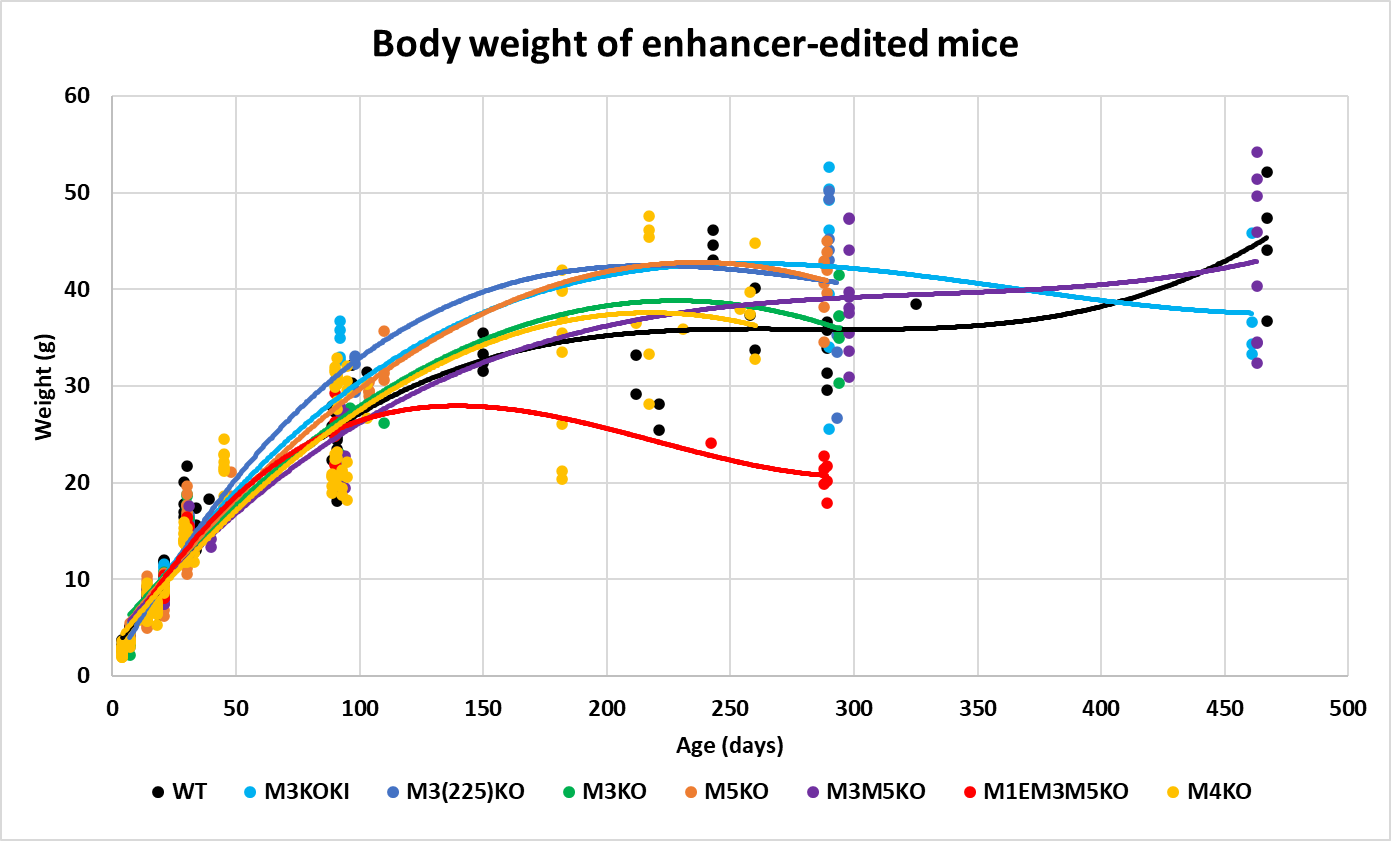


**Supplementary figure 6. The body weight of enhancer-edited mice during development and aging.**

**Supplementary table 1.** Relative *Mbp/Gapdh* mRNA in cervical spinal cord of enhancer-edited lines. The values are presented as mean % ± SEM. “*” and “**” represent P-values ≤ 0.05 and ≤ 0.01 respectively. n(F:M) represents the number of Female and Male mice from each genotype analyzed at each age.

**Supplementary table 2.** Relative *Golli/Gapdh* mRNA in cervical spinal cord of enhancer-edited lines. The values are presented as mean % ± SEM. “*” and “**” represent P-values ≤ 0.05 and ≤ 0.01 respectively. n(F:M) represents the number of Female and Male mice from each genotype analyzed at each age.
